# Ceftazidime/Tobramycin Co-Loaded Chitosan-Coated Zein Nanoparticles against Antibiotic-Resistant and Biofilm-Producing *Pseudomonas aeruginosa* and *Klebsiella pneumoniae*

**DOI:** 10.3390/ph17030320

**Published:** 2024-02-29

**Authors:** Luís André de Almeida Campos, Azael Francisco Silva Neto, Alexsandra Maria Lima Scavuzzi, Ana Catarina De Souza Lopes, Nereide Stela Santos-Magalhães, Isabella Macário Ferro Cavalcanti

**Affiliations:** 1 Biochemistry Sector, Keizo Asami Institute (iLIKA), Federal University of Pernambuco (UFPE), Recife 50670-901, PE, Brazil; luis.andre@ufpe.br (L.A.d.A.C.); azael.silvaneto@ufpe.br (A.F.S.N.); 2 Sector of Clinical Microbiology, Keizo Asami Institute (iLIKA), Federal University of Pernambuco (UFPE), Recife 50670-901, PE, Brazil; 3 Laboratory of Microbiology, Department of Tropical Medicine, Federal University of Pernambuco, Recife 50670-901, PE, Brazil; alexsandramariah@gmail.com (A.M.L.S.); ana.lopes.ufpe@gmail.com (A.C.D.S.L.); 4 Laboratory of Microbiology and Immunology, Academic Center of Vitória (CAV), Federal University of Pernambuco (UFPE), Vitória de Santo Antão 55608-680, PE, Brazil

**Keywords:** antibiotics, infections, nanocarriers, bacterial resistance, combination therapy

## Abstract

This study aimed to co-encapsulate ceftazidime and tobramycin in zein nanoparticles coated with chitosan and to characterize and evaluate the antibacterial and antibiofilm activity against antibiotic-resistant *Pseudomonas aeruginosa* and *Klebsiella pneumoniae*. Zein nanoparticles, synthesized using the nanoprecipitation method, were characterized by their particle size (Ø), polydispersity index (PDI), zeta potential (ζ), pH, and encapsulation efficiency (%EE). The chitosan coating provided stability, and physicochemical analyses revealed chemical interactions, efficient drug encapsulation, and thermal stability. The release kinetics demonstrated controlled release in simulated gastric and intestinal pH. The antibacterial activity, assessed by minimum inhibitory concentration (MIC) and minimum bactericidal concentration (MBC), indicated effectiveness against both pathogens. Antibiofilm assays, conducted using the crystal violet method, demonstrated the inhibition and eradication of biofilms. The chitosan-coated zein nanoparticles with CAZ and/or TOB exhibited Ø (315–335 nm), PDI (<0.2), ζ (+40 to +50 mV), pH (5), and %EE (>55%). Notably, the co-encapsulation formulation (CAZ–TOB–ZNP–CH) showed enhanced antibacterial and antibiofilm activities compared to the individual formulations. These findings suggest that the developed nanoparticles present a promising alternative for treating respiratory and intestinal infections caused by antibiotic-resistant and biofilm-producing *P. aeruginosa* and *K. pneumoniae*.

## 1. Introduction

Infections in the intestinal and respiratory tracts caused by antibiotic-resistant bacteria are among the top 10 leading causes of death and represent a challenge to public health and the global economy [1,2,3]. Intestinal infections caused more than 2.648 million deaths in 2020, while respiratory infections caused more than 3.051 million deaths, causing high levels of morbidity and hospitalization [1,4,5,6]. These infections have been of concern to healthcare professionals and the scientific community, especially nowadays, due to cases of resistant hospital-acquired infections and bacterial coinfection with SARS-CoV-2, the etiologic agent of COVID-19 [2,7].

The bacteria that cause these infections include *Pseudomonas aeruginosa* and *Klebsiella pneumoniae*. *P. aeruginosa* is an opportunistic Gram-negative pathogen that is associated with intestinal infections, pneumonia, and other diseases, such as irritable bowel syndrome or ulcerative colitis in hospitalized, immunocompromised patients. This bacterium is associated with structural lung diseases, such as cystic fibrosis, and is a common coinfectant in patients with COVID-19 [3,8,9].

*K. pneumoniae* is related to urinary tract infections, intestinal infections, and pneumonia, as well as infections affecting elderly patients with chronic diseases and/or respiratory diseases or the immunosuppressed. It causes secondary coinfection during hospitalization in patients with COVID-19, aggravating the clinical picture and causing longer hospital stays, poor prognosis, and mortality [8,10,11].

Infections caused by *P. aeruginosa* and *K. pneumoniae* are difficult to treat, especially when they are resistant to antimicrobials and biofilm producers [12,13]. These infections are common in hospitals, especially among long-term hospitalized patients exposed to invasive devices, thus requiring a strategic therapeutic regimen [3,14]. Furthermore, the ability of *P. aeruginosa* and *K. pneumoniae* to form biofilms is a major cause of therapeutic failure [9,13,14].

Research into new antibiotics is essential, due to the increase in bacterial resistance that limits the use of existing medicines and treatments. The infections caused by *K. pneumoniae* and *P. aeruginosa* are recognized worldwide for causing high rates of morbidity and mortality, especially when these infections are secondary and opportunistic [15,16]. To treat these infections, polymyxins, ceftazidime–avibactam, tigecycline, sulbactam and sulbactam-containing combinations, aminoglycosides, fosfomycin, and especially those combination therapies containing these antimicrobial agents, are used [17,18].

The diversity in response to treatment demonstrates the complexity of this scenario, mainly with infections promoted by bacteria with drug resistance profiles that may vary between patients, including resistance to carbapenems, fluoroquinolones, and other classes of antibiotics. Thus, bacterial resistance, the formation of biofilms, and the lack of correct treatment are the limitations of the use of conventional antibiotics, requiring new antibacterial therapeutic strategies in the treatment of serious infections [19,20].

**Figure 1:**
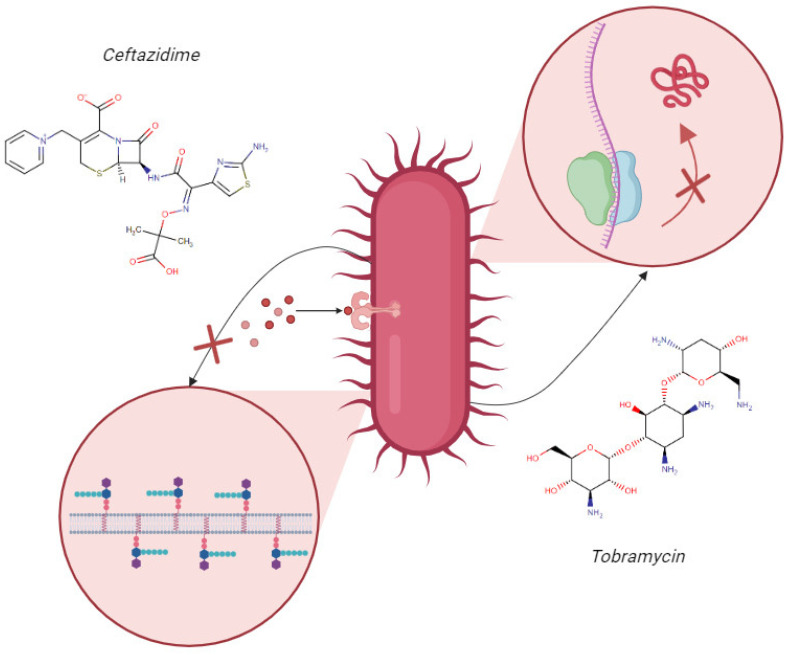
Scheme of the mechanisms of action of ceftazidime and tobramycin. This image illustrates the mechanism of action of the antibiotics ceftazidime and tobramycin. The arrows originate from the bacterial cell and point towards a zoomed-in view of the cell wall and the nucleus. The arrow pointing towards the cell wall indicates the inhibitory action of ceftazidime on cell wall synthesis, as represented by the “X”. The arrow pointing towards the nucleus indicates the inhibitory action of tobramycin on protein synthesis, also represented by the “X”. Created by the authors in Biorender.

Therefore, the use of combined antimicrobial therapy of an aminoglycoside, such as tobramycin (TOB), associated with a β-lactam, such as ceftazidime (CAZ), for treating infections caused by resistant strains and biofilm formers is an alternative option [10,21,22]. The therapeutic combination of ceftazidime and tobramycin has emerged as a promising strategy in the treatment of multidrug-resistant bacterial infections. This approach is widely used in clinical practice, due to its potential for synergistic interaction [23,24]. The synergistic potential is attributed to the distinct mechanisms of action of these two molecules. CAZ binds to bacterial cell wall proteins and inhibits bacterial cell wall synthesis, promoting the disruption of the cell wall, due to instability, and bacterial death (Figure 1). Meanwhile, TOB enters more easily into the bacterial cell, due to the instability of the cell wall, binding to the 30S subunit of the bacterial ribosome, interfering with protein synthesis, and causing bacterial death (Figure 1) [24,25]. 

Therefore, when combining ceftazidime and tobramycin, the synergistic potential of their complementary mechanisms of action favors the possibility of a more effective therapy against bacterial infections, especially in cases of multidrug resistance and in patients with cystic fibrosis [23,24,25]. However, these drugs are unstable, due to pH variation, are sensitive to degradation by digestive enzymes, and have low oral and nasal bioavailability, limiting their administration by these routes [21,22]. 

Encapsulating these drugs in chitosan-coated zein nanoparticles is a proposed antimicrobial therapy that can be used to overcome these limitations, increase bioavailability, and enable the delivery of the antimicrobials within biofilms [26,27]. Zein is a protein that forms a non-toxic, biocompatible, gastro-resistant, mucoadhesive, easily obtained, and low-cost polymer with hydrophilic and hydrophobic regions [27,28]. Chitosan is a natural polysaccharide derived from chitin that has non-toxic, biocompatible, mucoadhesive, biodegradable, and antimicrobial characteristics. Coating zein nanoparticles with this polymer can improve the stability and efficiency of the drug encapsulation, making it an advantageous alternative for the controlled release of antimicrobials [26,27,29]. 

Thus, this research presents an innovative character as it proposes, as goals, encapsulating and co-encapsulating CAZ and TOB in chitosan-coated zein nanoparticles for oral and nasal administration and evaluating the antibacterial activity of these nanocarriers against multidrug-resistant and biofilm-producing *P. aeruginosa* and *K. pneumoniae*.

## 2. Results 

### 2.1. Characterization of Nanoparticles

#### 2.1.1. Particle Size, Polydispersity Index, Zeta Potential, pH, and Encapsulation Efficiency of Ceftazidime and Tobramycin in Nanoparticles

The nanoparticles were characterized by their Ø, PDI, ζ, pH, and %EE (Table 1), and the DLS distribution curves are shown in Figure S1.

#### 2.1.2. Scanning Electron Microscopy, Fourier-Transform Infrared Spectroscopy, and X-ray Diffraction of the Nanoparticles

**Figure 2:**
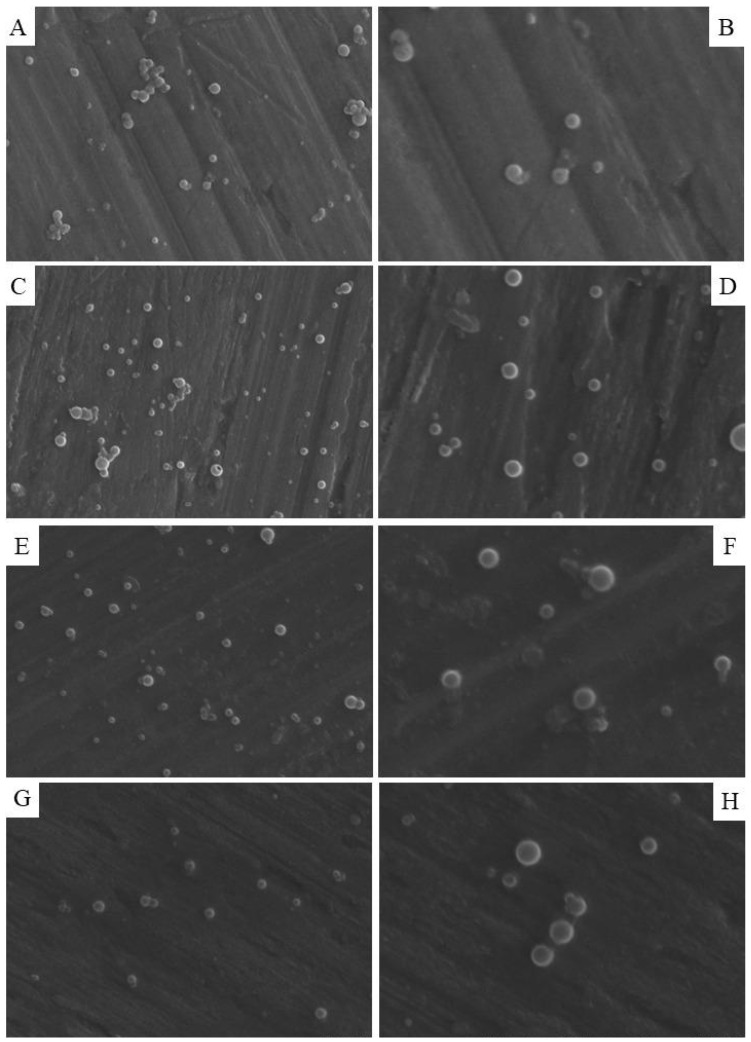
Morphology of ZNP–CH (**A**,**B**), CAZ–ZNP–CH (**C**,**D**), TOB–ZNP–CH (**E**,**F**), and CAZ–TOB–ZNP–CH (**G**,**H**) obtained from scanning electron microscopy. (**B**,**D**,**F**) (**right**) images are enlargements of images (**A**,**C**,**E**) (**left**), respectively.

The images of the nanoparticles obtained by SEM show spherical and smooth morphology with a homogeneous population of ZNP–CH (Figure 2A,B), CAZ–ZNP–CH (Figure 2C,D), TOB–ZNP–CH (Figure 2E,F), and CAZ–TOB–ZNP–CH (Figure 2G,H). The results evidenced in the distribution graphs prepared from the SEM images using ImageJ Version 1.54 and Origin 8 software corroborate with the results obtained by the dynamic light scattering technique carried out using Zetasizer Nano-ZS90 (Malvern, Worcestershire, UK), demonstrating that there is homogeneity in the average diameter of the particles, with a greater number of nanoparticles being in the range between 300 and 350 nm (Figures S1 and S2).

**Figure 3:**
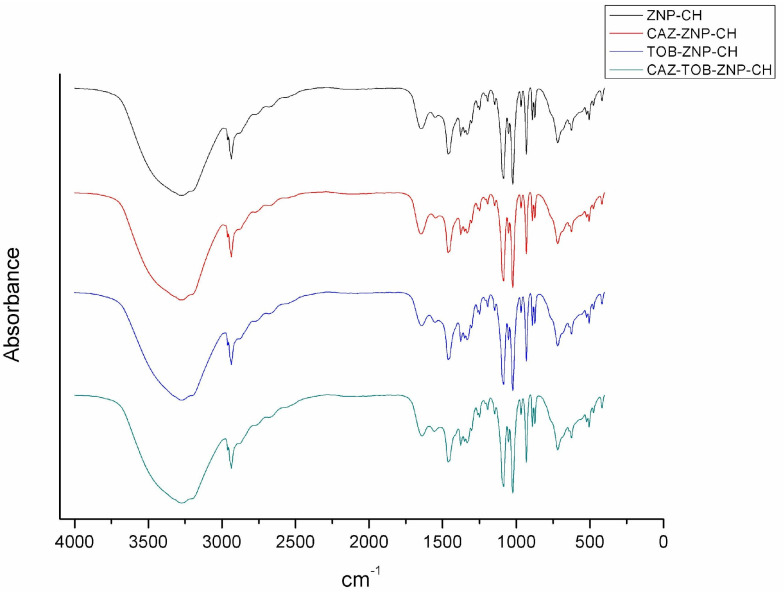
FTIR spectrum of chitosan-coated zein nanoparticles (ZNP–CH), chitosan-coated zein nanoparticles containing ceftazidime (CAZ–ZNP–CH), chitosan-coated zein nanoparticles containing tobramycin (TOB–ZNP–CH), and chitosan-coated zein nanoparticles containing ceftazidime and tobramycin (CAZ–TOB–ZNP–CH).

From the FTIR spectrum of ZNP–CH, CAZ–ZNP–CH, TOB–ZNP–CH, and CAZ–TOB–ZNP–CH (Figure 3), it can be seen for all of the formulations that the stretching vibration peak at around 3440 cm^−1^ corresponding to the hydroxyl groups of zein was suppressed due to the chemical interaction between the zein and chitosan polymers. 

Chitosan was identified in the FTIR spectrum by the presence of stretching at 3290 cm^−1^, referring to O–H and N–H bonds, stretching vibration at 2927–2864 cm^−1^, referring to the C–H bond, and stretching vibration at 1645 cm^−1^ and 1584 cm^−1^, referring to the amide I, amine, and amide II groups. Characteristic chitosan vibrations were observed in the ZNP–CH, CAZ–ZNP–CH, TOB–ZNP–CH, and CAZ–TOB–ZNP–CH spectra. However, the stretching vibration at 2864 cm^−1^ was unclear, and there was a stretching (C–N) shift from 1150 cm^−1^ to 1147 cm^−1^ and vibration from 896.73 cm^−1^ to 890.38 cm^−1^ in all of the nanoparticles.

In the CAZ spectrum, a band was observed in the region of 3000–3600 cm^−1^, referring to OH grouping, NH_2_, and a C–H bond, and stretching at 1810 cm^−1^, referring to the presence of a C–S–C bond. In Figure 3, CAZ–ZNP–CH and CAZ–TOB–ZNP–CH do not show stretching at 1810 cm^−1^, and there was a peak shift from 1670 cm^−1^ to 1645.55 cm^−1^ (CAZ–ZNP–CH) and 1639.47 cm^−1^ (CAZ–TOB–ZNP–CH) and from 1450 cm^−1^ to 1458 cm^−1^ for all of the CAZ–containing nanoparticles. 

The TOB spectrum, in turn, presents absorption bands at 3400–3200 cm^−1^ due to N–H or O–H stretching, at 2910 cm^−1^ due to aliphatic C–H stretching, at 1588 cm^−1^ due to N–H bending, at 1461 cm^−1^ due to CH_2_ grouping, at 1349–1380 cm^−1^ due to in-plane O–H bending vibration, and at 1032 cm^−1^ due to C–N or C–O stretching. Figure 3 shows a shift from 2910 cm^−1^ to 2936 cm^−1^ and from 1032 cm^−1^ to 1080 cm^−1^ in TOB–ZNP–CH and CAZ–TOB–ZNP–CH. Figure 3 shows the absorption band characteristics of zein, lysine, and chitosan in all of the nanoparticles; however, in the formulations containing drugs, there is a shift in the bands and an absence of other bands, indicating their interaction with the carrier polymer and the encapsulation of these molecules.

**Figure 4:**
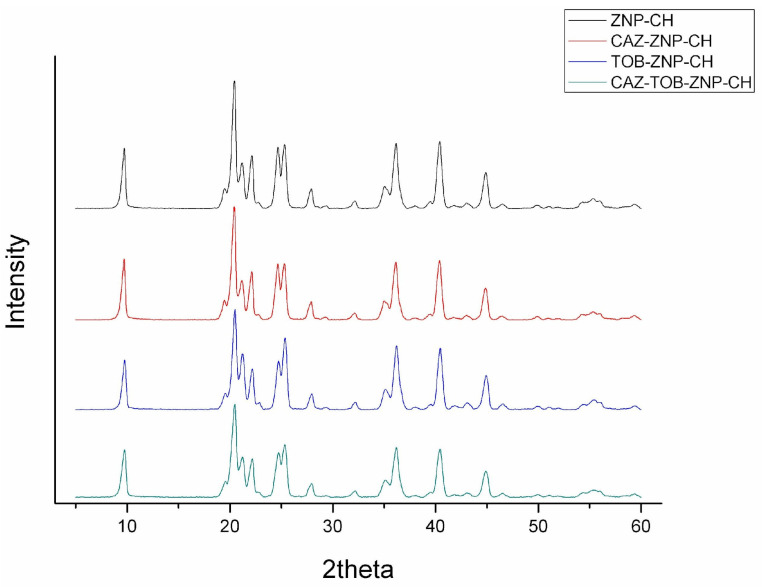
XRD of chitosan-coated zein nanoparticles (ZNP-CH), chitosan-coated zein nanoparticles containing ceftazidime (CAZ–ZNP–CH), chitosan–coated zein nanoparticles containing tobramycin (TOB–ZNP–CH), and chitosan-coated zein nanoparticles containing ceftazidime and tobramycin (CAZ–TOB–ZNP–CH).

Figure 4 highlights the XRD for ZNP–CH, CAZ–ZNP–CH, TOB–ZNP–CH, and CAZ–TOB–ZNP–CH. Three peaks (9.6°, 19.4°, and 20.4°) were observed in all of the nanoparticles. 

#### 2.1.3. Thermal Analysis by Thermogravimetry and Differential Scanning Calorimetry of the Nanoparticles

**Figure 5:**
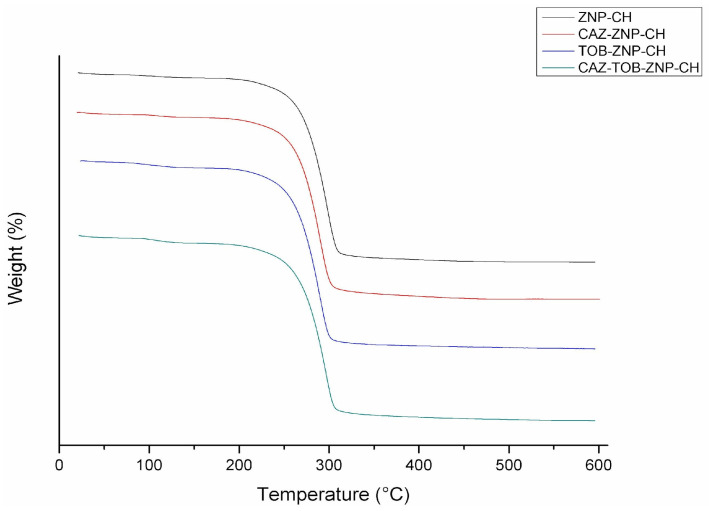
Thermogram of chitosan-coated zein nanoparticles (ZNP–CH), chitosan-coated zein nanoparticles containing ceftazidime (CAZ–ZNP–CH), chitosan-coated zein nanoparticles containing tobramycin (TOB–ZNP–CH), and chitosan-coated zein nanoparticles containing ceftazidime and tobramycin (CAZ–TOB–ZNP–CH).

The thermogravimetric curves obtained from the nanoparticles demonstrate an initial mass loss rate of up to 150 °C, around 2.5% for ZNP–CH and CAZ–ZNP–CH and around 4% for TOB–ZNP–CH and CAZ–TOB–ZNP–CH, and a loss of ≥90% in mass as the temperature increased from 200 °C to 325 °C (Figure 5). 

**Figure 6:**
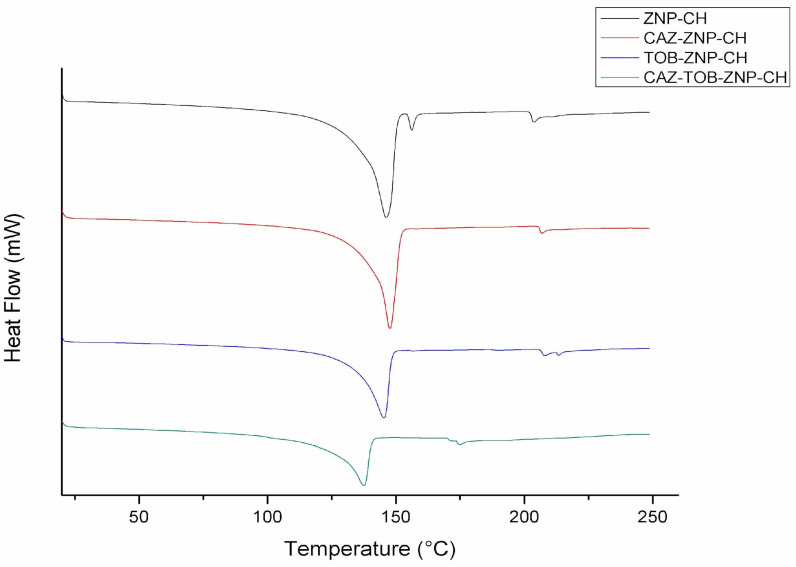
DSC of chitosan-coated zein nanoparticles (ZNP–CH), chitosan-coated zein nanoparticles containing ceftazidime (CAZ–ZNP–CH), chitosan-coated zein nanoparticles containing tobramycin (TOB–ZNP–CH), and chitosan-coated zein nanoparticles containing ceftazidime and tobramycin (CAZ–TOB–ZNP–CH).

In the DSC thermogram, the zein nanoparticles showed endothermic peaks, as follows: ZNP–CH at 146.17 °C, 156.18 °C, and 203.30 °C; CAZ–ZNP–CH at 147.66 °C and 206.33 °C; TOB–ZNP–CH at 145.34 °C and 204.23 °C; and CAZ–TOB–ZNP–CH at 137.56 °C and 174.21 °C (Figure 6). 

#### 2.1.4. Stability of CAZ–ZNP–CH, TOB–ZNP–CH, and CAZ–TOB–ZNP–CH under Simulated Gastrointestinal pH Conditions

**Figure 7:**
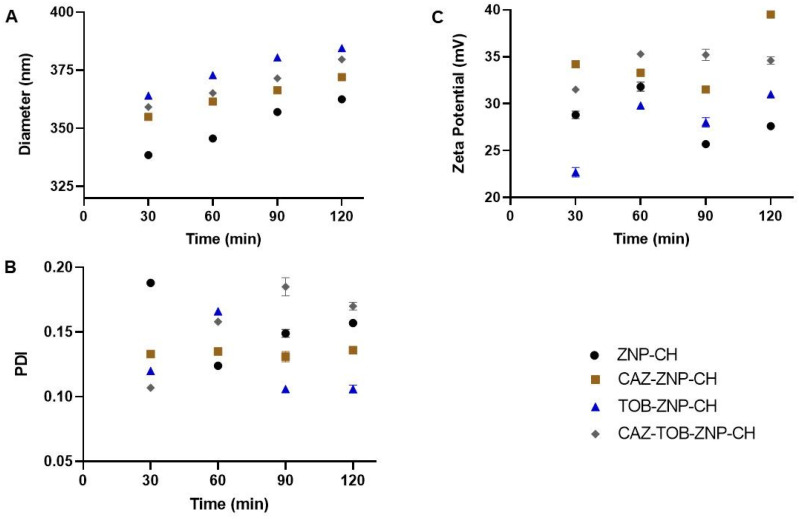
Average diameter (**A**), PDI (**B**), and zeta potential (**C**) of ZNP–CH, CAZ–ZNP–CH, TOB–ZNP–CH, and CAZ–TOB–ZNP–CH at pH 1.2. Legend: ZNP–CH: Chitosan-coated zein nanoparticles; CAZ–ZNP–CH: chitosan-coated zein nanoparticles containing ceftazidime; TOB–ZNP–CH: chitosan-coated zein nanoparticles containing tobramycin; and CAZ–TOB–ZNP–CH: chitosan-coated zein nanoparticles containing ceftazidime and tobramycin.

**Figure 8:**
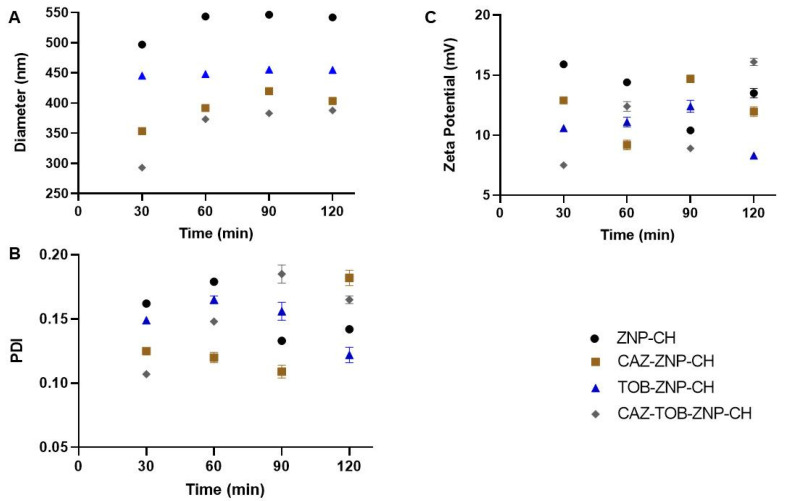
Average diameter (**A**), PDI (**B**), and zeta potential (**C**) of ZNP-CH, CAZ-ZNP-CH, TOB-ZNP-CH, and CAZ-TOB-ZNP-CH at pH 6.8. Legend: ZNP-CH: Chitosan-coated zein nanoparticles; CAZ-ZNP-CH: chitosan-coated zein nanoparticles containing ceftazidime; TOB-ZNP-CH: chitosan-coated zein nanoparticles containing tobramycin; and CAZ-TOB-ZNP-CH: chitosan-coated zein nanoparticles containing ceftazidime and tobramycin.

The nanoparticles were exposed to simulated gastric and intestinal media to assess their stability against pH variation. As observed in Figure 7A and Figure 8A, there was a gradual increase in the nanoparticles’ mean diameter over 120 min at pH 1.2 and pH 6.8. After this period, at pH 1.2 and 6.8, there was an increase of around 75 nm and 130 nm for all of the nanoparticles, respectively. All of the nanoparticles showed a decreased PDI value from 0.2 to 0.1 after 120 min at pH 1.2 and 6.8 (Figure 7B and Figure 8B). After the exposure to the simulated biological fluids, the zeta potential decreased gradually, at pH 1.2 and 6.8, reducing to around 15 mV and 25 mV for all of the nanoparticles, respectively (Figure 7C and Figure 8C).

#### 2.1.5. Long-Term Stability of Nanoparticles

The Ø, PDI, ζ, pH, and drug content of CAZ-ZNP-CH (Table 2), TOB-ZNP-CH (Table 3), and CAZ-TOB-ZNP-CH (Table 4) were evaluated for four months.

CAZ-ZNP-CH, TOB-ZNP-CH, and CAZ-TOB-ZNP-CH showed Ø of around 340 nm, PDI of around 0.2, ζ of around +40 mV, pH between 4.8 and 5.3, and CAZ and TOB content between 98 and 99%. These results point out that the Ø, PDI, ζ, pH, and drug content did not show significant changes during the 180 days, indicating that freeze-drying and storage conditions at 4 °C do not alter nanoparticles’ stability.

#### 2.1.6. In Vitro Release Kinetics of CAZ and TOB from Chitosan-Coated Zein Nanoparticles

**Figure 9:**
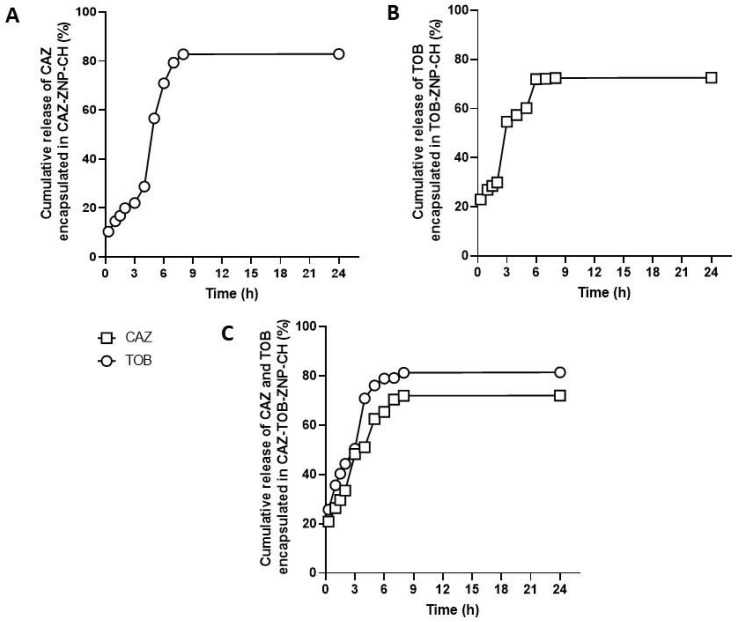
Release profile of ceftazidime from CAZ–ZNP–CH (**A**), of tobramycin from TOB–ZNP–CH (**B**), and ceftazidime and tobramycin from CAZ–TOB–ZNP–CH (**C**) in a gastrointestinal medium. Legend: CAZ: ceftazidime; TOB: tobramycin.

The results obtained from the release kinetics of CAZ encapsulated in CAZ-ZNP-CH and CAZ-TOB-ZNP-CH show a different kinetic profile in the simulated gastric (pH 1.2) and intestinal (6.8) medium. For the CAZ kinetics from CAZ-ZNP-CH, the release was observed at around 19% after exposure for 2 h at pH 1.2, at around 82% after exposure at pH 6.8, and, after 8 h, the controlled release of CAZ was maintained up to 24 h (Figure 9A).

For the CAZ release kinetics from CAZ-TOB-ZNP-CH, it was possible to observe a burst effect after 30 min with a release of around 21% at pH 1.2. After a 2 h exposure at this pH, 34% of CAZ was released, and, after 6 h at pH 6.8, 70% of CAZ was released, maintaining the controlled release of CAZ until 24 h of exposure (Figure 9C). 

Regarding the release of the TOB contained in TOB-ZNP-CH, a burst effect was noticed at around 23% after 30 min, then a 30% release after exposure for 2 h at pH 1.2, and around 72% release after exposure at pH 6.8, followed by a controlled release up to 24 h of exposure (Figure 9B). For the TOB kinetics from CAZ-TOB-ZNP-CH, it was possible to observe a burst effect after 30 min with 25% release, a 45% release after 2 h at pH 1.2, and around 81% release after exposure at pH 6.8, followed by a controlled release up to 24 h of exposure (Figure 9C). 

### 2.2. Microbiological Analyses

#### 2.2.1. Evaluation of Antibacterial Activity

The MIC of CAZ ranged from 12.5 to >50 µg/mL, and the MBC ranged from 25 to >50 µg/mL. For TOB, the MIC was 6.25 and 12.5 µg/mL, and the MBC was 12.5 and 50 µg/mL. For CAZ–ZNP–CH, the MIC was 3.12 and 12.5 µg/mL, and the MBC was 12.5 and 25 µg/mL. For TOB–ZNP–CH, the MIC was 1.56 and 3.12 µg/mL, and the MBC was 6.25 and 25 µg/mL. CAZ–TOB–ZNP–CH showed a MIC of 0.19 and 3.12 µg/mL for CAZ, and 0.15 and 2.40 µg/mL for TOB, and an MBC of 1.56 and 6.25 µg/mL for CAZ and 1.21 and 4.87 µg/mL for TOB. The MIC and MBC values found for CAZ–TOB–ZNP–CH were obtained according to the encapsulation efficiency of the drugs co-encapsulated in this nanoparticulate formulation (Table 5). ZNP–CH did not show any antibacterial activity against any of the tested strains (>50 µg/mL).

#### 2.2.2. Determination of Biofilm Production by Bacterial Isolates

Among the eight clinical bacterial isolates assessed in this study, two *P. aeruginosa* strains and four *K. pneumoniae* strains showed moderate or strong biofilm production (Table 6). 

#### 2.2.3. Evaluation of the Inhibition of Biofilm Formation

**Figure 10:**
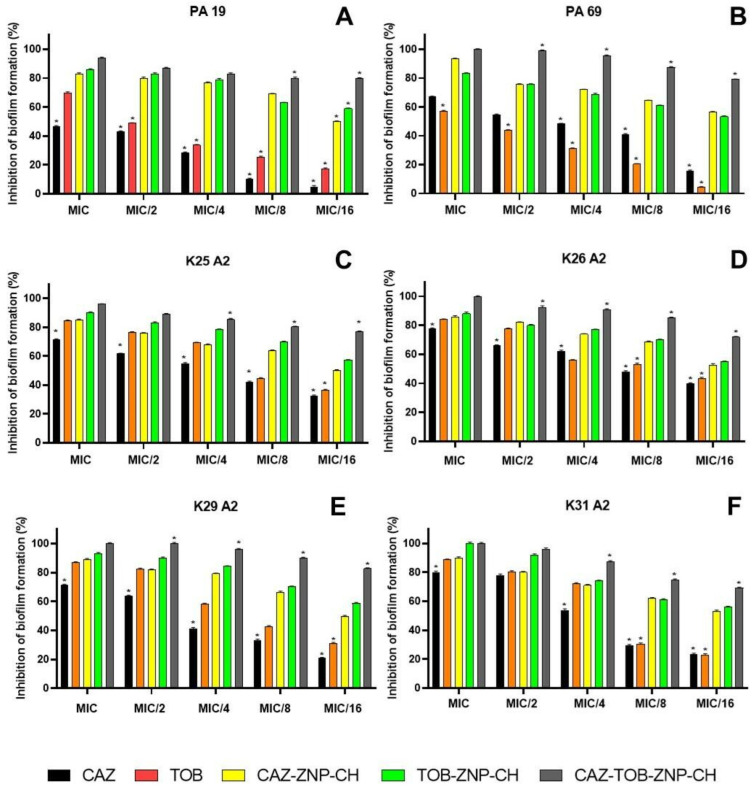
Inhibition of biofilm formation of clinical isolates of *Pseudomonas aeruginosa* (**A**,**B**) and *Klebsiella pneumoniae* (**C**–**F**) after treatment with ceftazidime and tobramycin encapsulated in chitosan–coated nanoparticles. The asterisk (*) indicates a statistically significant difference between treatments at the same concentration (*p* < 0.05). Legend: MIC: Minimum inhibitory concentration; CAZ: ceftazidime; TOB: tobramycin; CAZ–ZNP–CH: chitosan-coated zein nanoparticles containing ceftazidime; TOB–ZNP–CH: chitosan-coated zein nanoparticles containing tobramycin; CAZ–TOB–ZNP–CH: chitosan-coated zein nanoparticles containing ceftazidime and tobramycin.

The inhibition of biofilm formation by CAZ, TOB, CAZ–ZNP–CH, TOB–ZNP–CH, and CAZ–TOB–ZNP–CH occurred dose-dependently, with the highest inhibition at MIC and lowest inhibition at MIC/16 (Figure 10). Biofilm formation inhibition was observed between 5% and 80% for CAZ, between 4% and 88% for TOB, between 49% and 93% for CAZ–ZNP–CH, between 53% and 100% for TOB–ZNP–CH, and between 69% and 100% for CAZ–TOB–ZNP–CH (Figure 10). The CAZ and TOB encapsulated in the nanoparticles showed smaller MBIC values than the free drugs; moreover, CAZ–TOB–ZNP–CH exhibited 10- to 35-fold lower MBIC values than CAZ–ZNP–CH and TOB–ZNP–CH (Table 7). The MBIC values found for CAZ–TOB–ZNP–CH were obtained according to the encapsulation efficiency of the drugs co-encapsulated in this nanoparticulate formulation (Table 7). ZNP–CH did not inhibit biofilm formation at the concentrations tested for any of the strains used in this study. 

#### 2.2.4. Evaluation of Biofilm Eradication

**Figure 11:**
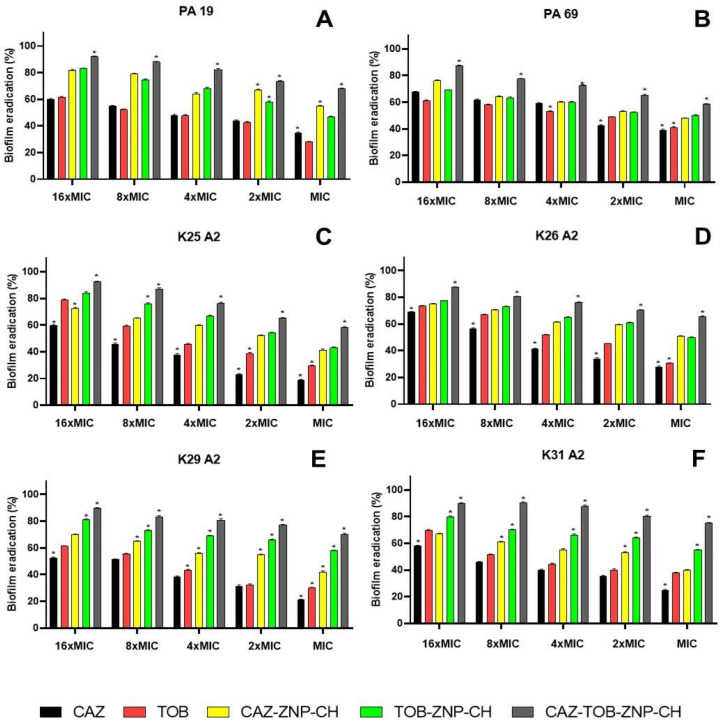
Eradication of biofilm produced by clinical isolates of *Pseudomonas aeruginosa* (**A**,**B**) and *Klebsiella pneumoniae* (**C**–**F**) after treatment with ceftazidime and tobramycin encapsulated in chitosan-coated nanoparticles. The asterisk (*) indicates a statistically significant difference between treatments at the same concentration (*p* < 0.05). Legend: MIC: Minimum inhibitory concentration; CAZ: ceftazidime; TOB: tobramycin; CAZ–ZNP–CH: chitosan-coated zein nanoparticles containing ceftazidime; TOB–ZNP–CH: chitosan-coated zein nanoparticles containing tobramycin; CAZ–TOB–ZNP–CH: chitosan-coated zein nanoparticles containing ceftazidime and tobramycin; PA: antimicrobial-resistant *Pseudomonas aeruginosa*; and K: antimicrobial-resistant *Klebsiella pneumoniae*.

Biofilm eradication by CAZ, TOB, CAZ–ZNP–CH, TOB–ZNP–CH, and CAZ–TOB–ZNP–CH also occurred dose-dependently, with the highest inhibition at 16×MIC and the lowest inhibition at MIC (Figure 11). Biofilm eradication was observed between 18% and 68% for CAZ, between 27% and 79% for TOB, between 40% and 81% for CAZ–ZNP–CH, between 43% and 84% for TOB–ZNP–CH, and between 58% and 92% for CAZ–TOB–ZNP–CH (Figure 11). The CAZ and TOB encapsulated in the nanoparticles showed smaller MBEC values than the free drugs; moreover, CAZ–TOB–ZNP–CH exhibited 8- to 300-fold lower MBIC values than CAZ–ZNP–CH and TOB–ZNP–CH (Table 8). The MBEC values found for CAZ–TOB–ZNP–CH were obtained according to the encapsulation efficiency of the drugs co-encapsulated in this nanoparticulate formulation (Table 8). ZNP–CH did not show antibiofilm activity at the concentrations tested for any of the strains used in this study.

## 3. Discussion

In this study, ZNP–CH, CAZ–ZNP–CH, TOB–ZNP–CH, and CAZ–TOB–ZNP–CH showed particle sizes smaller than 340 nm and PDI values smaller than 0.3. A small difference in the average diameter was observed between ZNP–CH, CAZ–ZNP–CH, TOB–ZNP–CH, and CAZ–TOB–ZNP–CH. As it is a nanosphere, chemical interaction with the encapsulated drugs can lead to the formation of more compact nanoparticles, changing the density and the size of the nanoparticles [30,31]. For this study, these small size variations are not relevant, since we designed these nanoparticles to be absorbed or bound to the mucosa, being developed as therapeutic strategies for oral and nasal applications [32,33]. 

A Ø value between 100 and 700 nm is ideal for nasal and oral applications, since NPs with this particle size can be transported intracellularly through the nasal epithelium [32], while NPs with sizes between 300 and 500 nm can be endocytosed in the intestine, mainly by M cells, which are specialized epithelial cells that line the lymphoid follicles of Peyer’s plates and can reach more specific biological targets due to their size and relative mobility in the intestine [33]. The PDI sets the homogeneity profile of dispersions, and values equal to or less than 0.3 indicate a monodisperse profile and are ideal for in vivo applications [30].

ZNP–CH, CAZ–ZNP–CH, TOB–ZNP–CH, and CAZ–TOB–ZNP–CH showed a positive surface charge with ζ above +35 mV. The ζ is a parameter that indicates nanoparticle stability, since values greater than +30 mV promote nanoparticle repulsion, minimizing the aggregation between them [33,34]. Positively charged nanocarriers exhibit interactions with airway mucosa, as strong electrostatic interactions occur with the sialic and acidic anionic parts of the glycosaminoglycans contained in mucin and on the surface of the airway cells [35,36]. Similarly, these positively charged surface-active nanostructures can prolong their residence time in the small intestine and exhibit strong adhesion to the intestinal mucosa due to their electrostatic interactions with negative charges in the mucus layer of this environment. Therefore, they may be more likely to be captured by Peyer’s patches [36,37].

Since parameters such as Ø, PDI, ζ, and pH are essential for determining the administration route, formulation stability, and applications for in vivo testing, the chitosan-coated zein nanoparticles developed in this study are suitable for administration through the nasal and oral routes. The pH values of the nanoparticles were around 5. The pH of the nasal fluid is slightly acidic, and is between 5.5 and 6.5, while the stomach pH can range between 1.5 and 2.0, and the intestinal pH between 6.0 and 8.0 [38,39].

The %EE of the CAZ in the nanoparticles ranged between 73.68 ± 0.7 and 87.28 ± 0.2%, and that of the TOB ranged between 57.47 ± 0.5 and 63.38 ± 0.7% (Table 1). The literature indicates a wide %EE variation of β-lactams and aminoglycosides in polymeric nanoparticles due to the chemical affinity of the drugs with the constituent polymers of NPs [39,40,41,42,43].

A reduction of approximately 10% occurred regarding the %EE of CAZ and TOB in CAZ–TOB–ZNP–CH. Drug co-encapsulation is a challenge, since encapsulating two drugs in the same nanoparticle can generate competition for binding sites [44,45]. However, co-encapsulation can allow for synergism, enhanced antimicrobial activity, a broadening of the antimicrobial spectrum, a decreased stimulation of resistance development, a relative dosage adjustment of multiple drugs, reduced toxic effects, and cost effectiveness [44,45].

Coating zein nanoparticles with carbohydrates increases encapsulation and improves the physical and photochemical stability of the drug, promoting greater molecular interaction between the drugs and their biological targets and facilitating the formation of nanoparticles with ideal physicochemical features for in vitro and in vivo applications [27,46,47]. Furthermore, chitosan has been shown to interact with the mucosal surfaces of the nasal, pulmonary, and gastrointestinal tracts [48,49]. Thus, its bioadhesiveness and resistance to degradation make this polymer convenient for coating particles for nasal and oral applications [29].

In the images, small aggregates of ZNP–CH and CAZ–ZNP–CH can be seen that do not interfere with the PDI of the formulations, as seen in Figure S2. It has been highlighted that no aggregates are observed in CAZ–TOB–ZNP–CH, this being the final formulation proposed in the treatment of *K. pneumoniae* and *P. aeruginosa* infections [31,50,51,52].

In the FTIR spectrum, absorption bands between 1600–1700 and 1510–1570 cm^−1^ representing the characteristics of amide I and amide II are present in all of the nanoparticles [53,54]. An absorption band ranging from 3500 to 3000 cm^−1^, referring to the NH^3+^ groups of the protonated side chain of amino acid lysine, was also observed in all of the nanoparticles [55]. It was also observed that symmetric C–H strain-related vibration at 1375 cm^−1^, the antisymmetric C–O–C stretching and C–N stretching at 1150 cm^−1^, the C–O stretching vibration at 1026 cm^−1^, and the peaks between 896 and 1154 cm^−1^ correspond to the saccharide structure of chitosan [56,57]. For CAZ, another stretching at around 1670 cm^−1^ was evident, due to the axial deformation of the amide group C=O at 1450 cm^−1^ by the C–N bond and between the bands 600–800 cm^−1^ by the adjacent hydrogen deformations [58,59]. The TOB spectrum, in turn, presents absorption bands at 3400–3200 cm^−1^, due to N–H or O–H stretching; at 2910 cm^−1^, due to aliphatic C–H stretching; at 1588 cm^−1^, due to N–H bending; at 1461 cm^−1^, due to CH2 grouping; at 1349–1380 cm^−1^, due to in-plane O–H bending vibration; and at 1032 cm^−1^, due to C–N or C–O stretching [60,61].

In the XRD figure, the peaks of 9.6° and 19.4° correspond to zein’s presence, indicating the molecule’s amorphous character [27,62]. However, through some intermolecular interactions with other compounds, one can have new diffraction of crystalline character [62]. The 20.4° peak corresponds to the chitosan presence, since two peaks are found in the diffractogram of this molecule at around 2θ = 10.5° and 20°, due to its high degree of crystallinity [63,64]. In Figure 4, other peaks (2θ = 21°, 22°, 24°, 25°, 36°, 40°, 43°, and 44°) of varying intensity were identified in the nanoparticles; however, they do not correspond to the characteristic peaks of CAZ (2θ at 20.2°, 21.5°, and 22.3°) [58,59,65,66] or TOB (2θ at 17, 7°, 18.3°, and 18.8°) [61,65], indicating only a small degree of crystallinity of these nanoparticles and possible hydrogen bonds or electrostatic interactions between the drugs, the carrier polymer, and the coating polymer.

In the TGA thermogram, this mass loss profile presented by the nanoparticles developed in this study is different from the zein degradation profile because, at 83.7 °C, there is a 5.6% loss, and, at 385.2 °C, there is a 73.8% loss [67]. Lysine has two weight losses in the temperature ranges of 255–365 °C (57%) and 478–589 °C (24%) [68], and chitosan presents three weight loss ranges, the first being between 50 and 100 °C (5%), the second between 280 and 390 °C (70%), and the third above 390 °C (15%) [69,70].

The zein mass loss is attributed to the breakdown of amino acid residues and the cleavage of the peptide bonds of the protein [67]. Meanwhile, chitosan mass loss occurs at the first point due to the evaporation of the water adsorbed to the chitosan polymer by hydrogen bonds, at the second by deacetylation depolymerization and cleavage of glycosidic bonds, and at the third by the decomposition of the residual carbon [69]. 

CAZ has mass loss at intervals of 50–100 °C of around 10%, and, above 190 °C, loss of around 75% occurs [71,72]. TOB shows a mass loss of around 90% between 25 °C and 125 °C [60,73]. The thermogram results show no significant mass loss between 25 °C and 200 °C, evidencing CAZ and TOB encapsulation and protection against heat degradation for the drugs and the thermal stability of the nanoparticles (Figure 5). In the DSC thermogram, zein demonstrates endothermic peaks in the range of 50–100 °C [74,75]. Chitosan, in turn, is characterized by an endothermic peak near 100 °C and an exothermic peak at around 270 °C [74,76]. Lysine shows two endothermic peaks at 74.1 °C and 259.3 °C [77,78]. CAZ shows an endothermic peak at around 120 °C [71], while TOB shows three endothermic peaks at 115.64 °C, 176.70 °C, and 230 °C, and an exothermic peak at 206.94 °C [65]. We can observe that the peaks corresponding to zein, lysine, and chitosan have been shifted, and the peaks corresponding to the drugs are not evident, indicating CAZ and TOB encapsulation in the nanoparticles and an improved thermal stability of these antibiotics. 

In a stable state at gastrointestinal pH, there was an increase in the nanoparticle size, a decrease in PDI, and a reduction in zeta potential at pH 1.2 and pH 6.8. Other studies in the literature have also proven the changes in these parameters after the exposure of chitosan-coated zein NPs to different pHs, such as the research of Wang et al. [79], who performed zein nanoparticle coating with carboxymethyl chitosan encapsulating β-carotene with antioxidant potential, and Khan et al. [80], who developed alginate/chitosan-coated zein nanoparticles containing resveratrol.

With values of pH below 6.0, chitosan undergoes a protonation process of the NH_2_ groups and, consequently, the expansion of the polymer chain and an increase in particle size [79,80]. Thus, it can be observed that the change in the diameter of the nanoparticles with the decrease in pH is related to the phenomenon of the protonation of the amino groups present in the molecular structure of chitosan [81,82]. The decrease in the zeta potential happens as an electrostatic effect of the adsorption of the other molecules and ions present in the simulated gastric fluid [76,83]. Furthermore, studies show that the presence of ions in the medium that are used to form gastric solutions, as mentioned above, can partially neutralize these positive charges, leading to less electrostatic repulsion between the particles and less aggregation [84,85].

When increasing the pH to 6.8, deprotonation of the ionized groups (-NH3+) occurs, and, thus, there is a reduction in the zeta potential, leading to less electrostatic repulsion between the nanoparticles and the promotion of colloidal aggregation and increased particle size [32,77]. The reduction in the surface charge may be an indication of nanoparticle destabilization, and, for application in intestinal infections, it is relevant that there is a destabilization and erosion of the nanoparticles in order to release the medicines into the intestinal region, promoting the fight against the resistant bacterial strains [82,83,85]. CAZ–ZNP–CH, TOB–ZNP–CH, CAZ–TOB–ZNP–CH, and ZNP–CH demonstrate colloidal stability by electrostatic and steric repulsion under simulated gastrointestinal conditions, demonstrating their potential for future applications as oral antimicrobial delivery vehicles for intestinal delivery.

As for stability in months, some studies have evaluated the stability of carbohydrate-coated zein nanoparticles. Yuan et al. [86], Cai et al. [87], and Zhang et al. [54] developed zein nanoparticles containing curcumin coated with dextran, pectin, and fucan, respectively. The authors evaluated the stability for 28 days and observed no significant macroscopic, particle size, or PDI changes. Other studies have already shown the long-term stability of chitosan-coated zein nanoparticles, such as Chen et al. [88], who encapsulated curcumin and piperine in chitosan-coated zein nanoparticles, and Xiao et al. [89], who produced zein nanoparticles associated with carboxymethyl chitosan containing genistein. These authors observed that Ø, PDI, and ζ showed no significant changes after a 60-day storage period. 

In the release kinetics tests, the results show that the formulations showed a kinetic profile with a rapid initial release, followed by a controlled release for up to 24 h in the simulated gastrointestinal fluids. The controlled release profile of antibiotics in simulated gastric and intestinal pH is influenced by coating with chitosan, as reported in the studies of Pauluk et al. [90], Chen et al. [91], Zhou et al. [92], and Ruan et al. [93], who encapsulated resveratrol, β-carotene, quercetin, and astilbine, respectively, in chitosan-coated zein nanoparticles. The chitosan coating allows for controlled drug release because it forms a thick, dense layer around the zein nanoparticles that promotes the slower release of the active ingredients [77,93]. 

*P. aeruginosa* and *K. pneumoniae* are bacteria that cause intestinal and respiratory infections affecting a large portion of the world’s population and are on the list of bacteria that pose the greatest risk to human health [94,95]. The *P. aeruginosa* isolates used in this study present a resistance to quinolones, polymyxins, cephalosporins, carbapenems, and other β-lactams [96]. The *K. pneumoniae* isolates used in this study carry the *bla*_KPC-2_ gene encoding the Ambler class A carbapenemase and the *acrB* and *acrF* genes encoding efflux pumps, conferring their resistance to carbapenems, cephalosporins, aminoglycosides, and quinolones [30]. The accumulation of various resistance mechanisms results in infections with high mortality rates due to the scarcity and inefficiency of therapies. Thus, is necessary to develop new therapeutic options [96,97]. Some studies have developed therapeutic strategies to combat bacterial infections, testing the antibacterial activity of CAZ or TOB encapsulated alone in nanocarriers, especially liposomes, against bacterial strains with and without an antibiotic resistance profile [98,99,100,101]. 

Torres et al. [98] encapsulated CAZ in liposomes (LIPO–CAZ) and tested the antimicrobial potential against *P. aeruginosa* strain SPM-1 (clinical isolate resistant to cefepime and ceftazidime). CAZ and LIPO–CAZ showed MICs of 1024 µg/mL and 512 µg/mL against *P. aeruginosa* SPM-1, respectively. Hedayati Ch et al. [101] tested the activity of TOB encapsulated in niosomes against *P. aeruginosa* strains resistant to β-lactams, aminoglycosides, and quinolones. The MIC values for the TOB ranged from 2 to 8 µg/mL, and, for the TOB-containing niosomes, the range was observed from 0.125 to 2 µg/mL. The MBC values varied from 2 to 8 µg/mL for the TOB and from 0.125 to 4 µg/mL for the niosomes containing TOB. 

The data show that the CAZ or TOB encapsulated in the nanocarriers exhibit antibacterial potential. However, the nanocarriers developed by Torres et al. [98] and Hedayati Ch et al. [95] are lipidic and without a polymeric coating; therefore, they are unstable, because they undergo rapid enzymatic digestion and bile salt actions that interact with the liposomes, such as surfactants in the gastrointestinal tract [102]. Thus, these liposomes cannot be administered orally, unlike CAZ–ZNP–CH and TOB–ZNP–CH.

Nevertheless, CAZ–TOB–ZNP–CH presented more in vitro antibacterial activity than CAZ–ZNP–CH or TOB–ZNP–CH, evidencing that antibiotic combination potentiates the antimicrobial effect. Some studies have performed the co-encapsulation of antibacterial agents and have evaluated their action. Schiffelers et al. [103] developed liposomes encapsulating gentamicin and ceftazidime (LE–GN–CZ), testing their antibacterial activity in vivo in mice with lung infections caused by resistant strains of *K. pneumoniae*. A single dose of LE–GN–CZ (2.5/1.6 mg/kg) applied for 14 days increased the animal survival rate compared to the LE–GN (20 mg/kg) and LE–CZ (12.5 mg/kg) formulations applied alone, showing that the synergistic interaction was effective in overcoming infections promoted by resistant *K. pneumoniae*. 

Ye et al. [99] encapsulated clarithromycin (CLA) and TOB in liposomes (TOB/CLA–CPROLips) and tested the antibacterial activity against *P. aeruginosa* strain PAO1. CLA, TOB, and TOB/CLA–CPROLips had MIC values of >16 µg/mL, 16 µg/mL, 1 µg/mL, respectively. Wang et al. [100] developed liposomes containing colistin and ciprofloxacin to treat infections caused by multidrug-resistant *P. aeruginosa* H131300444 and *P. aeruginosa* H133880624. The MIC value of colistin was 128 µg/mL against both of the strains, while ciprofloxacin was 16 µg/mL for *P. aeruginosa* H133880624 and 8 µg/mL for *P. aeruginosa* H131300444. The combination of the two drugs co-encapsulated in the liposomes at a concentration of 8 µg/mL eradicated the growth of both of the strains within 24 h.

The co-encapsulation of these drugs in the nanocarriers developed by Schiffelers et al. [97], Ye et al. [99], and Wang et al. [100] showed antibacterial potential, as well as CAZ–TOB–ZNP–CH. However, the use of CAZ–TOB–ZNP–CH has become promising for oral and nasal administration, unlike the liposomes developed by the above authors. 

In this study, we have observed the antibacterial potential of all of the formulations encapsulating CAZ and TOB, especially the formulation containing both drugs. This potentiation of antibacterial action happened because of the association of the two different action mechanisms, since CAZ acts by inhibiting the synthesis of the peptidoglycan of the bacteria cell wall and TOB induces the formation of non-functional proteins compromising the bacterial metabolism, leading to bacterial death [104,105].

Thus, combination therapy is considered an effective strategy to treat multidrug-resistant bacterial infections. The administration of combined drugs in a single vehicle enables the synergistic action of the different mechanisms of action, the delivery of the drugs to the infection sites, the proper exposure of patients to the drug, and the reduced stimulus for developing bacterial resistance [46,106], making co-encapsulation in chitosan-coated zein nanoparticles a promising alternative for resistant respiratory and intestinal infections. 

Biofilm is a virulence factor with clinical relevance, as it is associated with healthcare infections, causing concern for healthcare professionals and the general population [107]. Poor antibiotic penetration through the biofilm matrix and the presence of persistent cells contribute to the resistance of biofilm-forming bacteria to antibiotics, leading to persistent infections that cause hospitalization, patient suffering, and reduced quality of life [103,104]. Biofilms are constantly associated with human diseases, including surgical implant infections, gum disease, and digestive, urinary, and respiratory tract infections induced by catheters and other invasive devices [107,108]. The infections caused by these bacteria become serious when they colonize the GIT of hospitalized and immunocompromised patients [107,108,109]. Moreover, biofilms have been associated with the initiation and development of stomach, small intestine, and colon cancer by producing genotoxins. They are also associated with inflammatory bowel disease, especially the development of ulcerative colitis and Crohn’s disease caused by *P. Aeruginosa* and *K. pneumoniae* [110,111]. 

In the respiratory tract, *P. aeruginosa* produces biofilms in the sinuses, becoming a reservoir in lung abscesses in ventilator-associated pneumonia, in bronchiectasis, and in chronic lung infections associated with cystic fibrosis [112,113]. Meanwhile, *K. pneumoniae* is associated with biofilm production in pneumonia, promoting the pathogenicity and chronicity of respiratory infections [114,115]. The biofilm-producing ability of *P. aeruginosa* and *K. pneumoniae* is associated with increased morbidity and mortality in patients, especially those with other infections, such as COVID-19 [9,11]. Thus, a treatment with the potential to eliminate this microorganism, inhibit biofilm formation, and/or eradicate biofilms already formed is needed [107,108,109,110]. 

Biofilm production is an important factor in the survival and virulence of *P. aeruginosa* and *K. pneumoniae* in adverse environmental conditions, including hospital environments, especially in intensive care units (ICU) and surgical centers, facilitating the establishment and maintenance of chronic and persistent infections [116]. The aim of inhibiting biofilm formation has led to some studies proposing the co-encapsulation of antimicrobial agents in nanocarriers. Mahdiun et al. [117] encapsulated bismuth-ethanediol (BiEDT) and TOB in niosomes (Nio–BiEDT–TOB) and tested the antibiofilm activity at subinhibitory concentrations (MIC/2, MIC/4, MIC/8, and MIC/16) against *P. aeruginosa* ATCC 27853. The inhibition of BiEDT, TOB, and Nio–BiEDT–TOB against this bacterium ranged from 35% to 60%, 45% to 63%, and 45% to 80%, respectively. 

Ye et al. [99] encapsulated CLA and TOB in liposomes (TOB/CLA–CPROLips) and tested the inhibition of biofilm formation against *P. aeruginosa* strain PAO1. At subinhibitory concentrations, CLA, TOB, and TOB/CLA–CPROLips inhibited the biofilm formation by 2% to 5%, 5% to 15%, and 15% to 30%, respectively. The studies by Mahdiun et al. [111] and Ye et al. [99] show lower percentages of biofilm formation inhibition than those observed for CAZ–ZNP–CH, TOB–ZNP–CH, and CAZ–TOB–ZNP–CH, indicating that the encapsulation of CAZ and TOB alone, or in combination, in chitosan-coated zein nanoparticles have greater potential for antibiofilm activity and can be administered orally and nasally. 

The physicochemical properties of the formulations influence the antibiofilm activity. The coating of zein nanoparticles by chitosan imparts a positive surface charge onto CAZ–ZNP–CH, TOB–ZNP–CH, and CAZ–TOB–ZNP–CH, enabling the electrostatic interaction of these nanocarriers with the surface of the negatively charged bacterial cells. This interaction can reduce the adhesion of the bacteria to surfaces, preventing biofilm formation [118,119]. The literature has proven the antibiofilm activity of CAZ and TOB. CAZ reduces the expression of the *ibpA* gene, decreasing bacterial motility; reduces the expression of adhesion genes *fimG*, *csgA*, and *ybgD*; reduces the expression of the motility gene *flgA* and the genes regulating quorum sensing (QS); reduces the communication process between bacterial cells *luxS* and *luxR*; increases the expression of the indole synthesis gene *tnaA*, negatively modulating biofilm production; and inhibits the production of bis-(3′,5′)-cyclic dimeric guanosine monophosphate (c-di-GMP), which promotes the biosynthesis of exopolysaccharides, such as Pel and alginate, thereby negatively modulating biofilm formation [120,121,122]. As for TOB’s antibiofilm activity, this drug suppresses the gene expression of *pelA* and *pslA*, genes encoding synthesis of the exopolysaccharides Pel and Psl, which promote the attachment of bacteria to surfaces, thus inhibiting biofilm formation [101]. Thus, this study suggests that chitosan-coated zein nanoparticles containing CAZ and/or TOB inhibit biofilm formation by the electrostatic interaction of the nanoparticle surface with the bacterial surface and by modulating the gene expression promoted by the drugs.

The biofilms produced are associated with antibacterial therapy failure, especially in healthcare-related infections (HAIs), causing longer hospital stays, high morbidity and mortality rates, and economic burden; therefore, treating these infections is a challenge, due to the scarcity of drugs that can eradicate biofilms [123,124]. 

From the perspective of eradicating biofilms, Halwani et al. [125] developed liposomes encapsulating bismuth-thiol and tobramycin (LipoBiEDT–TOB) and evaluated the antibiofilm activity against aminoglycoside-resistant *P. aeruginosa* strains (PA-48912-1, PA-4892-2, and PA-48913) isolated from patients with cystic fibrosis. The formulations encapsulating the antimicrobials in isolation and with free drugs did not eradicate biofilms at the tested concentrations; however, LipoBiEDT–TOB showed MBEC values of 64 µg/mL for PA-48912-1, 256 µg/mL for PA-4892-2, and 512 µg/mL for PA-48913. A study by Halwani et al. [125] showed no MBEC values for the formulations encapsulating only one drug, unlike the results for CAZ–ZNP–CH and TOB–ZNP–CH (12.5 to 50 µg/mL), which eradicated the biofilm. Moreover, the MBEC values of CAZ–TOB–ZNP–CH show that this formulation has a higher potential to eradicate biofilms than the formulation developed by Halwani et al. [125].

Some of the physicochemical aspects of the formulations, such as the particle size and zeta potential, are critical for biofilm eradication. The size of the nanoparticles collaborates in the penetration of the drugs through the exopolysaccharide matrix, since NPs between 10 and 500 nm penetrate through the water channels and biofilm pores [126,127]. In the present study, the average diameter of the nanoparticles ranged from 314 to 336 nm. 

In this study, the zeta potential of the nanoparticles ranged between +39 and +50 mV. The surface charge of the nanoparticles is another important parameter for penetrating biofilms, as positively charged nanocarriers are more attracted to biofilm surfaces (negative charge) and are more likely to penetrate and accumulate drugs inside biofilms, possibly eradicating them [126,128]. 

Furthermore, to eradicate biofilms, the direct interaction of nanocarriers with bacterial cells in the biofilm and/or bacteria detaching from the polymeric matrix, the interaction or denaturation of the EPS matrix, and cell death induction by action of antimicrobials become essential [120,126,127]. Thus, the chitosan-coated zein nanoparticles containing CAZ and TOB developed in this study are candidates for therapies for infections caused by biofilm-forming *P. aeruginosa* and *K. pneumoniae* [108,128,129].

## 4. Material and Methods

### 4.1. Preparation of Nanoparticles

The chitosan-coated zein nanoparticles (ZNP–CH) were prepared via the nanoprecipitation method adapted from Moreno et al. [130] and Park, Park, and Kim [131]. Initially, zein (200 mg) and lysine (20 mg) were solubilized in 25 mL of 70% ethanol for 1 h. Subsequently, the drip method was used to add 25 mL of ultrapure water to the zein and lysine suspension. Then, ethanol was evaporated with a rotary evaporator, and 25 mL of dispersion was obtained. To obtain the chitosan-coated zein nanoparticles, a 0.5% chitosan solution in a 5:1 (*v*/*v*) zein nanoparticles/chitosan ratio was added to the dispersion under magnetic stirring for 1 h. After this step, the ZNP–CH was lyophilized with 5% mannitol. 

The chitosan-coated zein nanoparticles containing ceftazidime (CAZ–ZNP–CH), tobramycin (TOB–ZNP–CH), and ceftazidime and tobramycin together (CAZ–TOB–ZNP–CH) were also synthesized following the methods of Moreno et al. [130] and Park, Park, and Kim [131], as described above. CAZ (12.5 mg) was solubilized in 5 mL of 70% ethanol solution, and TOB (12.5 mg) was solubilized in 2 mL of an aqueous solution for drug encapsulation. Then, the CAZ and/or TOB solutions were added to the zein/lysine solution under magnetic stirring for 1 h. Finally, CAZ–ZNP–CH, TOB–ZNP–CH, and CAZ–TOB–ZNP–CH were also lyophilized using 5% mannitol. 

### 4.2. Characterization of Nanoparticles

#### 4.2.1. Particle Size, Polydispersity Index, Zeta Potential, and Nanoparticle pH 

The particle size of CAZ–ZNP–CH, TOB–ZNP–CH, CAZ–TOB–ZNP–CH, and ZNP–CH was measured using the Zetasizer Nano-ZS90 (Malvern, Worcestershire, UK). The measurements were taken at 25 °C with a fixed angle of 90°, and the results were expressed as the mean hydrodynamic diameter (nm). A total of 50 µL of the nanoparticles were diluted in 950 µL of ultra-purified water (Milli Q^®^, Millipore, Danvers, MA, USA) for particle size analysis. The surface charge of the nanoparticles was set by defining the zeta potential (ζ). For the ζ measurement, 50 µL of the samples was diluted in 950 µL of purified water using the Zetasizer Nano-ZS90 (Malvern, Worcestershire, UK). At room temperature, the nanoparticles’ pH was measured with a glass electrode and an MS Tecnopon digital pH meter (mPA-210P, São Paulo, Brazil). The nanoparticles were kept lyophilized at 4 °C in a hermetically sealed glass tube. The results correspond to the independent experiments performed in triplicate on different days [132].

#### 4.2.2. Encapsulation Content and Efficiency of Ceftazidime and Tobramycin in Nanoparticles

An aliquot (350 µL) of CAZ–ZNP–CH, TOB–ZNP–CH, and CAZ–TOB–ZNP–CH was diluted in 1000 µL of 70% ethanol to determine the CAZ and TOB content in the nanoparticles, and methanol was added to complete in the 5000-µL volumetric flask. The CAZ in the nanoparticles was established by high-performance liquid chromatography with a UV/Vis detector (HPLC-UV/Vis) at 254 nm. As a stationary phase, a C18 column (250 mm × 4.6 mm, 5 μm, Xbridge Waters) was used, and the mobile phase used a solution of Acetonitrile: Milli-Q Water (2:98 *v*/*v*). An isocratic elution condition was used at a flow rate of 1 mL/min with a six min run time [98]. Derivatization was performed for samples with TOB using a 0.5% (*w*/*v*) fluorescamine solution in methanol under incubation at 24 °C protected from light for 1 h [65]. The TOB in the nanoparticles was determined at 390 nm using an Ultrospec 3000 pro spectrophotometer (Biochrom, Cambridge, UK).

The CAZ and TOB encapsulation efficiency (%EE) test was performed using the ultrafiltration/ultracentrifugation technique with filtration units (Amicon Ultra Centrifugal Filters; Millipore, Billerica, MA, USA). The formulation samples (500 µL) were inserted into the filters and subjected to ultracentrifugation at 8000 rpm for 1 h. A total of 350 µL of the filtered sample was diluted in 1000 µL of 70% ethanol, and methanol was added to the 5000-µL volumetric flask. The filtered CAZ was measured with HPLC-UV/Vis, and the filtered TOB was measured using an Ultrospec 3000 pro spectrophotometer, as described previously. Independent experiments were performed in triplicate for each condition on different days. The encapsulation efficiency was determined following the equation below:

$$\mathrm{EE}=\frac{Total\;amount\;of\;CAZ\;or\;TOB-unloaded\;amount\;CAZ\;or\;TOB\times 100}{Total\;amount\;of\;CAZ\;or\;TOB}$$

#### 4.2.3. Scanning Electron Microscopy, Fourier-Transform Infrared Spectroscopy, and X-ray Diffraction of Nanoparticles

The scanning electron microscopy (SEM) technique was used for nanoparticle morphological analysis. CAZ–ZNP–CH, TOB–ZNP–CH, CAZ–TOB–ZNP–CH, and ZNP-CH were diluted in ultrapure water at a ratio of 1:10 *v*/*v* nanoparticles:water. The nanoparticles were spread on stubs and kept in an oven at 37 °C for 24 h. After drying, the stubs were metalized (FINE COAT, ION SPUTTER JFC-1100) and observed under a ZEISS scanning electron microscope, model EVO-LS15 [133]. 

The FTIR spectra of CAZ–ZNP–CH, TOB–ZNP–CH, CAZ–TOB–ZNP–CH, and ZNP–CH were obtained by mixing freeze-dried samples with potassium bromide (KBr) pellets. The samples were scanned from 4000 to 400 cm^−1^ in the Fourier-transform spectrophotometer (IR-TF) (Frontier/PerkinElmer model, Waltham, MA, USA), obtaining spectra at 4 cm^−1^ resolution [91].

The XRD analysis of CAZ–ZNP–CH, TOB–ZNP–CH, CAZ–TOB–ZNP–CH, and ZNP–CH was performed using an X-ray diffractometer (Rigaku, model Miniflex, Cedar Park, TX, USA). The data were collected over an angular range from 5° to 50° 2-theta in continuous mode using a step size of 0.02° 2-theta and step time of 5 s [134].

#### 4.2.4. Thermal Analysis by Thermogravimetry and Differential Scanning Calorimetry of Nanoparticles

The TGA curves of CAZ–ZNP–CH, TOB–ZNP–CH, CAZ–TOB–ZNP-CH, and ZNP-CH were obtained using TGA Q500 equipment (TA Instruments, Boston, MA, USA). The samples (5 mg) were heated in a platinum tray at a 10 °C/min rate, with a nitrogen flow rate of 20 mL/min, from temperatures of 25 to 600 °C [133]. 

The DSC curves of CAZ–ZNP–CH, TOB–ZNP–CH, CAZ–TOB–ZNP–CH, and ZNP–CH were obtained using a DSC Q10 (TA Instruments, Boston, MA, USA). Each analytical sample (5 mg) was weighed onto an aluminum tray, and then the tray was sealed. The samples were heated from 20 to 250 °C at a constant heating rate of 10 °C/min and a flow rate of 20 mL/min of nitrogen gas [131].

#### 4.2.5. Stability of CAZ–ZNP–CH, TOB–ZNP–CH, and CAZ–TOB–ZNP–CH under Simulated Gastrointestinal pH Conditions 

The stability of the nanoparticles was evaluated in simulated gastric and intestinal pH biological media solutions, which were prepared according to Cavalcanti et al. [132]. Initially, 250 μL of CAZ–ZNP–CH, TOB–ZNP–CH, or CAZ–TOB–ZNP–CH was added into 750 μL of gastric (pH 1.2) or intestinal (pH 6.8) solution in microtubes, and the microtubes were constantly stirred (70 rpm) at 37 °C for 2 h. At 30 min intervals, aliquots were withdrawn and evaluated for Ø, PDI, and ζ.

#### 4.2.6. Long-Term Stability of Nanoparticles 

CAZ–ZNP–CH, TOB–ZNP–CH, and CAZ–TOB–ZNP–CH were kept lyophilized at 4 °C for four months and evaluated monthly to determine the Ø, PDI, ζ, and drug content.

#### 4.2.7. In Vitro Release Kinetics of CAZ and TOB from Chitosan-Coated Zein Nanoparticles

The in vitro release kinetic profile of CAZ and TOB was evaluated using the dialysis technique (cellulose membrane, cut-off 15–20 kD, Sartorius, Göttingen, Germany) in different solutions to simulate the pH of the gastrointestinal tract (pH 1.2 and pH 6.8) and in saline-phosphate buffer (PBS) at pH 7.4 to simulate blood. The release kinetics assay was performed in a sink regime, where the volume of the dissolution medium was ten times that of the saturation volume. The solutions were prepared based on the methodology of Rezaei and Nasirpour [135], as described by the authors, as follows: The gastric solution was prepared by dissolving 2.0 g of sodium chloride in 80 mL of 1 M hydrochloric acid, and then water was added up to 1000 mL. The pH of this solution was 1.2. Purified pepsin was added to obtain the final gastric solution. The phosphate buffer was prepared by dissolving 6.8 g of monobasic potassium phosphate in 250 mL of water and then adding 77 mL of 0.2 M sodium hydroxide and water up to 1000 mL. The final pH was adjusted to 6.8 using 0.2 N sodium hydroxide or 0.2 N hydrochloric acid. Purified pancreatin was added to obtain the final phosphate solution.

The membrane was hydrated, and an aliquot (3 mL) of CAZ–ZNP–CH, TOB–ZNP–CH, or CAZ–TOB–ZNP–CH dispersions was inserted into the membrane dialysis bag and placed in a tube containing 30 mL of pH 1.2 solution under stirring conditions (70 rpm) at 37 °C for 2 h. At 30 min intervals, aliquots of 3 mL were withdrawn, and the same volume was replaced with a pH 1.2 solution. After 2 h, the formulation membrane was removed and inserted into another tube containing 30 mL of pH 6.8 solution. The agitation was maintained at 70 rpm at 37 °C, removing 3-mL aliquots after 1, 2, 3, 4, 5, 6, and 24 h and replacing the aliquots with the same volume of pH 6.8 solution, as performed by Cavalcanti et al. [132]. 

A 3-mL aliquot of CAZ–ZNP–CH, TOB–ZNP–CH, or CAZ–TOB–ZNP–CH was inserted into the membrane and placed in a tube containing 30 mL of buffer solution (pH 7.4) and kept under stirring conditions (70 rpm) at 37 °C for 42 h. Initially, the 3-mL aliquots were removed at 15 min, 30 min, and 45 min intervals up to the first hour. After the first hour, the aliquots were removed after 1, 2, 3, 4, 5, 6, 7, 8, 9, 10, 11, 12, 13, 14, 15, 16, 24, 30, 36, 40, and 42 h. As the 3-mL aliquots were removed, the same volume was inserted with PBS buffer solution of pH 7.4 for each aliquot removed. The CAZ and TOB were quantified with HPLC-UV/Vis and a spectrophotometer, respectively, as described previously.

### 4.3. Microbiological Analyzes

#### 4.3.1. Obtaining Clinical Bacterial Isolates

The clinical isolates used in this research were collected from hospitals in Recife-PE, Brazil. The *P. aeruginosa* strains were from a study by Costa-Júnior et al. [96] and the *K. pneumoniae* strains were from a study by Scavuzzi et al. [136]. The results of the characterization of the isolates using the VITEK2^®^ automated system (bioMérieux, Lyon, RO, France) and the polymerase chain reaction (PCR) to identify the genes related to the resistance mechanisms are described in Table 9. The strains were transported to the Clinical Microbiology Sector of the Keizo Asami Immunopathology Laboratory of the Federal University of Pernambuco (LIKA/UFPE), where they were kept in glycerol at −80 °C. 

#### 4.3.2. Evaluation of Antibacterial Activity 

According to the Clinical and Laboratory Standards Institute, the in vitro antibacterial activity of the drugs encapsulated in the nanoparticles was evaluated using the broth microdilution method [137]. The clinical isolates of *P. aeruginosa* (PA 19, PA 56, and PA 69) and *K. pneumoniae* (K25 A2, K26 A2, K29 A2, K31 A2, and K32) were used for this assay. Müeller–Hinton broth (MHC) was initially dispensed into each well of the plates. CAZ, TOB, CAZ–ZNP–CH, TOB–ZNP–CH, and CAZ–TOB–ZNP–CH were added through serial dilution at concentrations of 0.097 to 50 µg/mL, and, lastly, the bacterial suspensions were added at a final concentration of 10^5^ CFU/mL. MHC without inoculum was used as a sterility control (negative control), MHC with inoculum was used as a microbial growth control (positive control), and ZNP–CH was used as an experimental negative control. The microplates were incubated at 35 ± 2 °C for 24 h, and the minimum inhibitory concentration value (MIC) was determined as the lowest concentration capable of inhibiting 90% of bacterial growth by spectrophotometry at a wavelength of 630 nm. The minimum bactericidal concentration (MBC) was determined after the MIC results. A sample aliquot from the wells with no visible growth was inoculated onto Müeller–Hinton agar, and the plates were incubated at 35 ± 2 °C for 24 h. After this period, the MBC was determined as the lowest concentration with no microbial growth [137]. Independent experiments were performed in triplicate on different days.

#### 4.3.3. Determination of Biofilm Production by Bacterial Isolates

Biofilm-producing bacterial isolates were identified using the crystal violet method [138]. Initially, tryptone soy broth (TSB) + glucose (1%) was dispensed into each well of the microdilution plates. Then, bacterial suspensions (10^5^ CFU/mL) of the clinical isolates of *P. aeruginosa* (PA 19, PA 56, and PA 69) and *K. pneumoniae* (K25 A2, K26 A2, K29 A2, K31 A2, and K32) were added and incubated at 35 ± 2 °C for 24 h. After incubation, the wells’ contents were aspirated and washed with pH 7.4 phosphate buffer. The plates were dried, and then the adhered bacteria were fixed with 99% methanol. After fixation, the methanol was removed, and the plates were put to dry again. Afterward, the bacteria adhered to the plates were stained with 1% crystal violet. Excess dye was removed, and 30% glacial acetic acid was added to each well. Then, an analysis of the result was performed via spectrophotometry at 570 nm (Multiskan FC microplate photometer, Thermo Scientific, Madrid, Spain). The wells containing only culture medium were used as negative controls. The strains were classified into four categories based on the OD (optical densities) values of the bacterial biofilms compared to ODc (optical density of the control) value, as follows: non-adherent if OD ≤ Odc; weak biofilm production if Odc < OD ≤ 2 × Odc; moderate biofilm production if 2 × Odc < OD ≤ 4 × Odc; or strong biofilm production if 4 × Odc < OD [138]. Independent experiments were performed in triplicate on different days.

#### 4.3.4. Evaluation of Inhibition of Biofilm Formation 

The assays used to evaluate the inhibition of biofilm formation were performed on the strains that proved to be moderate or strong biofilm producers. Initially, TSB + glucose (1%) was distributed in each well of the microdilution plates. CAZ, TOB, CAZ–ZNP–CH, TOB–ZNP–CH, and CAZ–TOB–ZNP–CH were added at concentrations of MIC, MIC/2, MIC/4, MIC/8, and MIC/16, and then bacterial suspensions (10^5^ CFU/mL) of the clinical isolates of *P. aeruginosa* (PA 19 and PA 69) and *K. pneumoniae* (K25 A2, K26 A2, K29 A2, and K31 A2) were added. The microplates were incubated at 35 ± 2 °C for 24 h. TSB without inoculum was used as a sterility control (negative control), TSB with inoculum was used as a microbial growth control (positive control), and ZNP–CH was used as an experimental negative control. After incubation, the inhibition of biofilm production was quantified using the crystal violet method [138], and the results were expressed as the minimum biofilm inhibitory concentration (MBIC) and an inhibition percentage [139]. Independent experiments were performed in triplicate for each condition on different days. The results of this test were analyzed in GraphPad Prism 5.0 software (GraphPad, San Diego, CA, USA) and expressed as mean ± standard deviation (SD).

#### 4.3.5. Evaluation of Biofilm Eradication 

The assays used to evaluate biofilm eradication were performed with the same strains as those previously studied. Initially, bacterial inoculum of the clinical isolates of *P. aeruginosa* (PA 19 and PA 69) and *K. pneumoniae* (K25 A2, K26 A2, K29 A2, and K31 A2) were adjusted to a density of 0.5 of the McFarland scale in TSB + glucose (1%) and distributed on microdilution plates. The plates were incubated at 35 ± 2 °C for 24 h to allow biofilm formation. After incubation, the culture medium was removed, and the medium was replenished. CAZ, TOB, CAZ–ZNP-CH, TOB–ZNP–CH, and CAZ–TOB–ZNP–CH were dispensed onto microplates at concentrations of 16 × MIC, 8 × MIC, 4 × MIC, 2 × MIC, and MIC, which were incubated at 35 ± 2 °C for 24 h. TSB without inoculum was used as a sterility control (negative control), TSB with inoculum was used as a microbial growth control (positive control), and ZNP-CH was used as an experimental negative control. After incubation, biofilm eradication was quantified using the crystal violet method [138], and the results were expressed as the minimum biofilm eradication concentration (MBEC) and an inhibition percentage [139]. Independent experiments were performed in triplicate for each condition on different days. The results were analyzed in GraphPad Prism 5.0 software (GraphPad, CA, USA) and expressed as mean ± standard deviation (SD).

#### 4.3.6. Statistical Analysis

In the microbiological tests used to compare the means of multiple groups, one-way analysis of variance (ANOVA) was applied using Tukey’s multiple comparison procedure in GraphPad Prism 5.0 software (GraphPad, CA, USA). The statistical data were considered significant with *p* < 0.05.

## 5. Conclusions

In this study, zein nanoparticles coated with chitosan co-encapsulating ceftazidime and tobramycin were developed using the nanoprecipitation method. The nanoparticles presented characteristics suitable for oral and nasal administration for future applications in antibacterial therapy in vivo, such as a particle size between 300 and 350 nm, a positive surface charge, high encapsulation efficiency of drugs, spherical and smooth morphology, thermal and physicochemical stability for four months and in simulated gastric and intestinal pH, and controlled release for 24 h. The CAZ and TOB encapsulated in the nanoparticles showed in vitro antibacterial activity against the antibiotic-resistant clinical isolates of *P. aeruginosa* and *K. pneumoniae* and potentiated biofilm formation inhibition and biofilm eradication. Thus, the nanoparticles developed in this study, using a simple, low-cost, and scalable technique, are a promising therapeutic alternative for intestinal and respiratory infections caused by antibiotic-resistant and biofilm-producing *K. pneumoniae* and *P. aeruginosa*.

## Figures and Tables

**Table 1:** Characterization of ZNP–CH, CAZ–ZNP–CH, TOB–ZNP–CH, and CAZ–TOB–ZNP–CH.

	Ø (nm)	PDI	ζ (mV)	pH	%EE (CAZ)	%EE (TOB)
ZNP–CH	336.5 ± 0.5	0.214 ± 0.9	+50.7 ± 1.5	5.0	-	-
CAZ–ZNP–CH	314.6 ± 0.2	0.220 ± 1.0	+39.2 ± 0.7	5.0	87.28 ± 0.2	
TOB–ZNP–CH	317.7 ± 0.6	0.274 ± 0.7	+45.1 ± 1.0	5.0		63.38 ± 0.7
CAZ–TOB–ZNP–CH	330.6 ± 0.6	0.217 ± 1.1	+42.3 ± 1.2	5.0	73.68 ± 0.7	57.47 ± 0.5

Ø: Particle size; PDI: polydispersity index; ζ: zeta potential; %EE: encapsulation efficiency; ZNP-CH: chitosan-coated zein nanoparticles; CAZ-ZNP-CH: chitosan-coated zein nanoparticles containing ceftazidime; TOB-ZNP-CH: chitosan-coated zein nanoparticles containing tobramycin; CAZ–TOB–ZNP–CH: chitosan-coated zein nanoparticles containing ceftazidime and tobramycin.

**Table 2:** Long-term stability of chitosan-coated zein nanoparticles containing ceftazidime.

Months				
Parameters	1	2	3	4
Ø (nm)	330.5 ± 0.1	328.2 ± 0.5	332.9 ± 0.3	337.1 ± 0.9
PDI	0.220 ± 0.7	0.230 ± 0.2	0.243 ± 0.7	0.239 ± 0.4
ζ (mV)	+37.1 ± 0.2	+37.1 ± 0.3	+36.6 ± 0.2	+38.9 ± 0.8
pH	5.0	5.2	5.1	5.2
Drug content (%)	99.01 ± 0.3	99.1 ± 0.2	98.99 ± 0.3	99.2 ± 0.1

Ø: Particle size; PDI: polydispersity index; ζ: zeta potential.

**Table 3:** Long-term stability of chitosan-coated zein nanoparticles containing tobramycin.

Months				
Parameters	1	2	3	4
Ø (nm)	329.1 ± 0.3	328.8 ± 0.8	331.2 ± 0.7	329.4 ± 1.0
PDI	0.274 ± 0.5	0.272 ± 0.6	0.288 ± 0.5	0.282 ± 0.3
ζ (mV)	+44.3 ± 0.2	+42.9 ± 0.1	+43.5 ± 0.4	+42.3 ± 1.3
pH	4.9	5.0	5.2	5.2
Drug content (%)	98.64 ± 0.1	99.01 ± 0.2	98.92 ± 0.5	98.57 ± 0.3

Ø: Particle size; PDI: polydispersity index; ζ: zeta potential.

**Table 4:** Long-term stability of chitosan-coated zein nanoparticles containing ceftazidime and tobramycin.

Months				
Months	1	2	3	4
Ø (nm)	340.1 ± 0.4	342.3 ± 0.6	343.4 ± 0.8	347.2 ± 0.5
PDI	0.217 ± 0.2	0.222 ± 0.1	0.228 ± 0.6	0.229 ± 0.4
ζ (mV)	+40.2 ± 1.0	+38.1 ± 0.3	+39.2 ± 0.7	+40.9 ± 0.8
pH	5.1	5.1	5.2	5.0
CAZ content (%)	99.36 ± 0.1	99.19 ± 0.4	99.6 ± 0.5	98.71 ± 0.6
TOB content (%)	99.19 ± 0.2	99.1 ± 0.9	99.03 ± 0.6	98.99 ± 0.4

Ø: Particle size; PDI: polydispersity index; ζ: zeta potential; CAZ: ceftazidime; TOB: tobramycin.

**Table 5:** Antibacterial activity of ceftazidime and tobramycin encapsulated in chitosan–coated zein nanoparticles.

	PA 19		PA 56		PA 69		K25 A2		K26 A2		K29 A2		K31 A2		K32 A2	
	MIC	MBC	MIC	MBC	MIC	MBC	MIC	MBC	MIC	MBC	MIC	MBC	MIC	MBC	MIC	MBC
	µg/mL															
CAZ	50	>50	25	50	12.5	>50	50	>50	50	>50	50	>50	>50	>50	>50	>50
TOB	6.25	12.5	6.25	25	6.25	12.5	12.5	50	12.5	12.5	12.5	12.5	12.5	50	12.5	50
CAZ–ZNP–CH	6.25	25	3.12	12.5	3.12	12.5	6.25	25	6.25	12.5	6.25	12.5	3.12	12.5	12.5	25
TOB–ZNP–CH	1.56	12.5	1.56	6.25	3.12	12.5	1.56	6.25	3.12	6.25	1.56	6.25	1.56	25	3.12	6.25
CAZ–TOB–ZNP–CH	0.39/0.30	1.56/1.21	0.19/0.15	1.56/1.21	0.78/0.60	3.12/2.40	0.78/0.60	3.12/2.40	1.56/1.21	3.12/2.40	0.39/0.30	1.56/1.21	1.56/1.21	6.25/4.87	3.12/2.40	6.25/4.87

MIC: Minimum inhibitory concentration; MBC: minimum bactericidal concentration; CAZ: ceftazidime; TOB: tobramycin; ZNP–CH: chitosan-coated zein nanoparticles; CAZ–ZNP–CH: chitosan-coated zein nanoparticles containing ceftazidime; TOB–ZNP–CH: chitosan-coated zein nanoparticles containing tobramycin; CAZ–TOB–ZNP–CH: chitosan-coated zein nanoparticles containing ceftazidime and tobramycin. PA: antimicrobial-resistant *Pseudomonas aeruginosa*; K: antimicrobial-resistant *Klebsiella pneumoniae*.

**Table 6:** Classification of biofilm production of antibiotic-resistant clinical isolates of *K. pneumoniae* and *P. aeruginosa*.

Bacterial Strain	Biofilm Production Classification
K 25 A2	Strong
K 26 A2	Moderate
K 29 A2	Moderate
K 31 A2	Moderate
K 32 A2	Weak
PA 19	Strong
PA 56	Weak
PA 69	Moderate

PA: Antimicrobial-resistant *Pseudomonas aeruginosa*; K: antimicrobial-resistant *Klebsiella pneumoniae*.

**Table 7:** Minimum inhibitory concentration of biofilm inhibition of ceftazidime and tobramycin encapsulated in chitosan-coated zein nanoparticles.

MBIC (µg/mL)						
AMOSTRAS	PA 19	PA 69	K A2 25	K A2 26	K A2 29	K A2 31
CAZ	>50	>12.5	50	50	50	>50
TOB	>6.25	>6.25	3.12	6.25	6.25	6.25
CAZ–ZNP–CH	0.781	0.781	1.56	1.56	1.56	1.56
TOB–ZNP–CH	0.195	0.39	0.39	0.39	0.195	0.39
CAZ–TOB–ZNP–CH	0.024/0.018	0.048/0.037	0.048/0.037	0.097/0.075	0.024/0.018	0.097/0.075

MBIC: Minimum biofilm inhibitory concentration; CAZ–ZNP–CH: chitosan-coated zein nanoparticles containing ceftazidime; TOB–ZNP–CH: chitosan-coated zein nanoparticles containing tobramycin; CAZ–TOB–ZNP–CH: chitosan-coated zein nanoparticles containing ceftazidime and tobramycin; PA: antimicrobial-resistant *Pseudomonas aeruginosa*; K: antimicrobial-resistant *Klebsiella pneumoniae*.

**Table 8:** MBEC of ceftazidime and tobramycin encapsulated in chitosan-coated zein nanoparticles.

MBEC (µg/mL)						
AMOSTRAS	PA 19	PA 69	K A2 25	K A2 26	K A2 29	K A2 31
CAZ	>800	>200	>800	>800	>800	>800
TOB	>100	>100	200	200	>200	>200
CAZ–ZNP–CH	50	50	50	100	100	50
TOB–ZNP–CH	12.5	50	12.5	25	12.5	12.5
CAZ–TOB–ZNP–CH	0.78/0.60	3.12/2.40	1.56/1.20	6.24/4.84	0.39/0.30	1.56/1.21

MBEC: Minimum biofilm eradication concentration; CAZ–ZNP–CH: chitosan-coated zein nanoparticles containing ceftazidime; TOB–ZNP–CH: chitosan-coated zein nanoparticles containing tobramycin; CAZ–TOB–ZNP–CH: chitosan-coated zein nanoparticles containing ceftazidime and tobramycin; PA: antimicrobial-resistant *Pseudomonas aeruginosa*; K: antimicrobial-resistant *Klebsiella pneumoniae*.

**Table 9:** Description of the phenotypic and genetic characteristics of *P. aeruginosa* and *K. pneumoniae* isolates.

Strain ID	Species	Antimicrobial Resistance	Resistance Genes	Possible Mechanism of Resistance to Beta-Lactams	Ref.
PA19	*P. aeruginosa*	MEM; IPM ATM; LVX; POL B	ND	ampC production, mutation, or loss of porins or increased expression of efflux pumps	[29]
PA56	*P. aeruginosa*	IPM; LVX; POL B	ND	ampC production, mutation, or loss of porins or increased expression of efflux pumps	[29]
PA69	*P. aeruginosa*	MEM; IPM ATM; TZP; CIP; FEP; LVX	ND	ampC production, mutation, or loss of porins or increased expression of efflux pumps	[29]
K25 A2	*K. pneumoniae*	AMC; AMO; ATM, CAZ, CFO; FEP; CTX; CIP, LEV; NAL; NOR; ERT; MEM; TZP; SUT	*bla*_KPC_; *acrB* and *acrF*	Serine-beta-lactamases and expression of efflux pumps	[30]
K26 A2	*K. pneumoniae*	AMC; ATM, CAZ, CFO; FEP; CTX; CIP, LEV; NAL; NOR; TZP; SUT	*bla*_KPC_; *acrB* and *acrF*	Serine-beta-lactamases and expression of efflux pumps	[30]
K29 A2	*K. pneumoniae*	AMC; ATM, CAZ, CFO; FEP; CTX; CIP, LEV; NAL; NOR; ERT; IPM; MEM; TZP; SUT	*bla*_KPC_; *acrB* and *acrF*	Serine-beta-lactamases and expression of efflux pumps	[30]
K31 A2	*K. pneumoniae*	AMC; ATM, CAZ, CFO; FEP; CTX; CIP, LEV; NAL; NOR; ERT; IPM; TZP; SUT	*bla*_KPC_; *acrB* and *acrF*	Serine-beta-lactamases and expression of efflux pumps	[30]
K32 A2	*K. pneumoniae*	AMC; ATM, CAZ, CFO; FEP; CTX; CIP, LEV; NAL; NOR; ERT; IPM; MEM; TZP; SUT	*bla*_KPC_; *acrB* and *acrF*	Serine-beta-lactamases and expression of efflux pumps	[30]

AMC: Amoxicillin/clavulanic acid; AMK: amikacin; GEN: gentamicin; TOB: tobramycin; MEM: meropenem; IPM: imipenem; ATM: aztreonam; TZP: piperacillin-tazobactam; LVX: levofoxacin; CIP: ciprofoxacin; FEP: cefepime; POL B: polymyxin B; CFO: cefoxitin; CTX: cefotaxime; CAZ: ceftazidime; NAL: nalidixic acid; NOR: norfloxacin; ERT: ertapenem; SUT: sufamethoxazole/trimethoprim.

## Data Availability

All data generated or analyzed during this study are included in this published article.

## Supplementary Materials

The following supporting information can be downloaded at: https://www.mdpi.com/article/10.3390/ph17030320/s1. Figure S1. The DLS distribution curves for ZNP–CH, CAZ–ZNP–CH, TOB–ZNP–CH and CAZ–TOB–ZNP–CH in Table 1; Figure S2. Particle size distribution with Average size of ZNP–CH (A), CAZ–ZNP–CH (B), TOB–ZNP–CH (C), and CAZ–TOB–ZNP–CH (D) from SEM images of nanoparticles. Ø: particle size; nm: nanometer.

## Abbreviation

AMK	Amikacin
AMC	Amoxicillin/Clavulanic Acid
K	Antimicrobial-Resistant *Klebsiella Pneumoniae*
PA	Antimicrobial-Resistant *Pseudomonas Aeruginosa*
ATM	Aztreonam
FEP	Cefepime
CTX	Cefotaxime
CFO	Cefoxitin
CAZ	Ceftazidime
CH	Chitosan
ZNP-CH	Chitosan-Coated Zein Nanoparticles
CAZ-ZNP-CH	Chitosan-Coated Zein Nanoparticles Containing Ceftazidime
CAZ-TOB-ZNP-CH	Chitosan-Coated Zein Nanoparticles Containing Ceftazidime and Tobramycin
TOB-ZNP-CH	Chitosan-Coated Zein Nanoparticles Containing Tobramycin
CIP	Ciprofoxacin
DSC	Differential Scanning Calorimetry
%EE	Encapsulation Efficiency
ERT	Ertapenem
FTIR	Fourier-Transform Infrared Spectroscopy
GEN	Gentamicin
HPLC	High-Performance Liquid Chromatography
IPM	Imipenem
LVX	Levofoxacin
MEM	Meropenem
MBC	Minimum Bactericidal Concentration
MBEC	Minimum Biofilm Eradication Concentration
MBIC	Minimum Biofilm Inhibitory Concentration
MIC	Minimum Inhibitory Concentration
NAL	Nalidixic Acid
nm	Nanometer
NOR	Norfloxacin
OD	Optical Densities
Ø	Particle Size
TZP	Piperacillin-Tazobactam
PDI	Polydispersity Index
PCR	Polymerase Chain Reaction
POL B	Polymyxin B
rpm	Revolutions Per Minute
PBS	Saline-Phosphate Buffer
SEM	Scanning Electron Microscopy
SUT	Sufamethoxazole/Trimethoprim
TGA	Thermogravimetric Analysis
TOB	Tobramycin
TOB	Tobramycin
TSB	Tryptone Soy Broth
XRD	X-ray Diffraction
ZNP	Zein Nanoparticles
ζ	Zeta Potential
