# Supplementary material for: Ceftazidime/Tobramycin Co-Loaded Chitosan-Coated Zein Nanoparticles against Antibiotic-Resistant and Biofilm-Producing Pseudomonas aeruginosa and Klebsiella pneumoniae
Source: Pharmaceuticals (Basel). 2024 Feb 29;17(3):320. doi: 10.3390/ph17030320 (PMC10974368; doi:10.3390/ph17030320)

Supplementary Material

Figure S1. The DLS distribution curves for ZNP−CH, CAZ−ZNP−CH, TOB−ZNP−CH and CAZ−TOB−ZNP−CH in table 1.

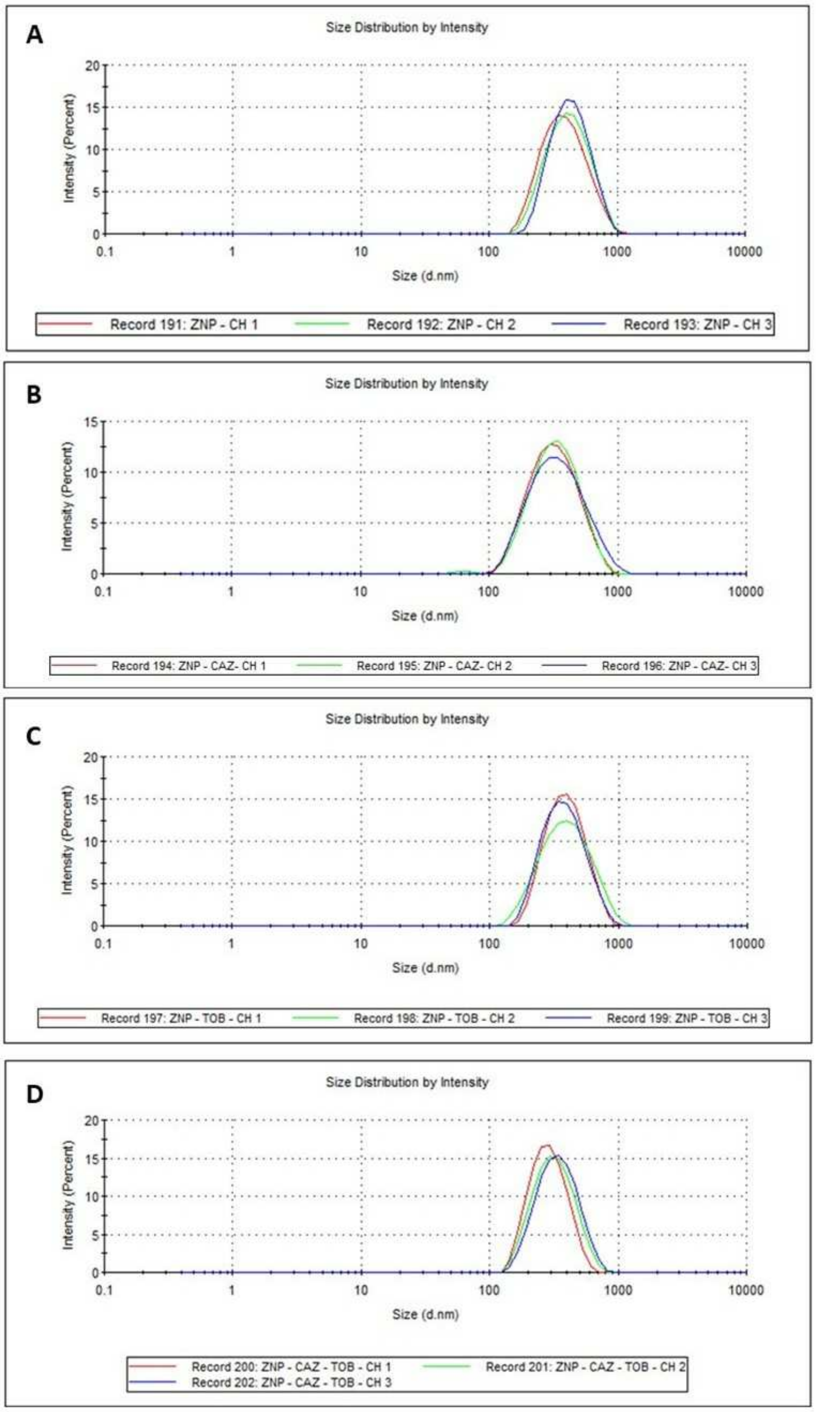

Figure S2. Particle size distribution with Average size of ZNP−CH (A), CAZ−ZNP−CH (B), TOB−ZNP−CH (C), and CAZ−TOB−ZNP−CH (D) from SEM images of nanoparticles. Ø: particle size; nm: nanometer.

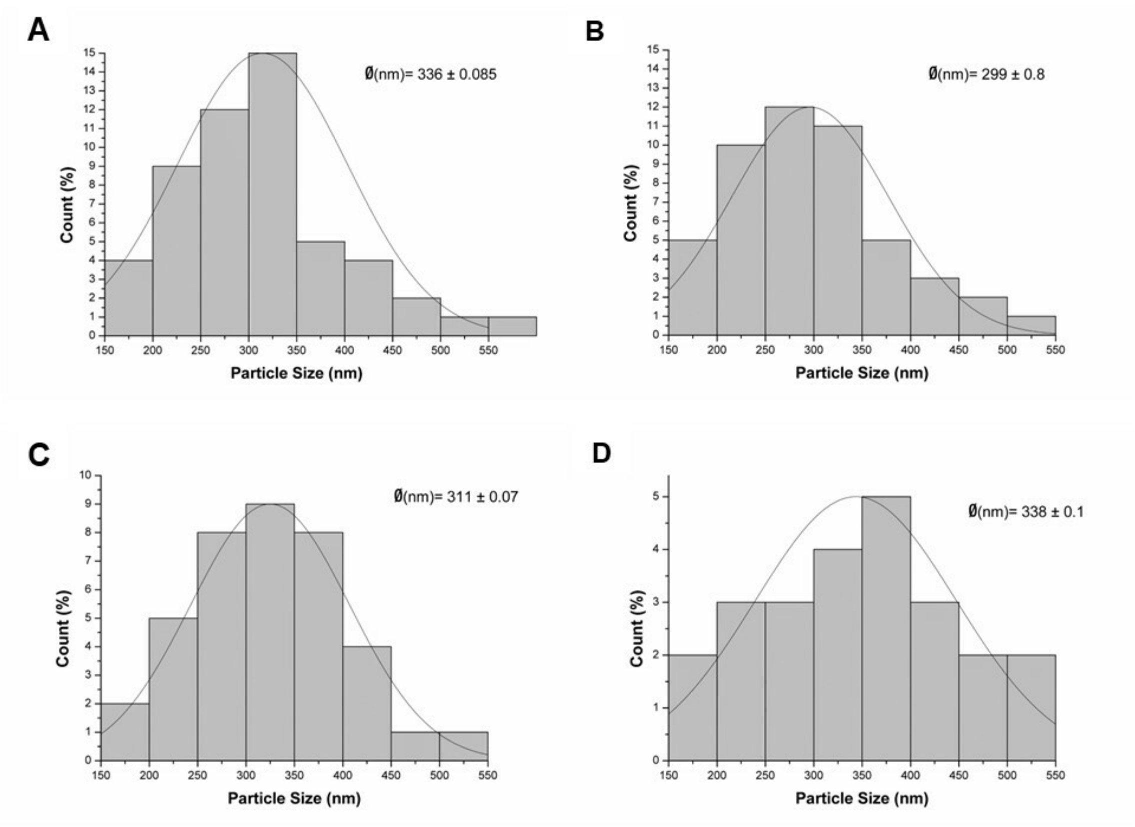

Supplement: Supplementary file 1 [file pharmaceuticals-17-00320-s001.zip › pharmaceuticals-2877090-supplementary.pdf]
